# ADNP promotes neural differentiation by modulating Wnt/β-catenin signaling

**DOI:** 10.1038/s41467-020-16799-0

**Published:** 2020-06-12

**Authors:** Xiaoyun Sun, Xixia Peng, Yuqin Cao, Yan Zhou, Yuhua Sun

**Affiliations:** 1 The Innovation of Seed Design, Chinese Academy of Sciences, Wuhan, 430072 China; 2 University of Chinese Academy of Sciences, Beijing, 100049 China; 3 Medical Research Institute, Frontier Science Center for Immunology and Metabolism, Wuhan University, Wuhan, 430071 China; 4 The Key Laboratory of Aquatic Biodiversity and Conservation, Institute of Hydrobiology, Chinese Academy of Sciences, Wuhan, 430072 China

**Keywords:** Cell signalling, Developmental neurogenesis, Stem-cell differentiation

## Abstract

ADNP (Activity Dependent Neuroprotective Protein) is a neuroprotective protein whose aberrant expression has been frequently linked to neural developmental disorders, including the Helsmoortel-Van der Aa syndrome (also called the ADNP syndrome). However, its role in neural development and pathology remains unclear. Here, we show that ADNP is required for neural induction and differentiation by enhancing Wnt signaling. Mechanistically, ADNP functions to stabilize β-Catenin through binding to its armadillo domain which prevents its association with key components of the degradation complex: Axin and APC. Loss of ADNP promotes the formation of the degradation complex and β-Catenin degradation via ubiquitin-proteasome pathway, resulting in down-regulation of key neuroectoderm developmental genes. In addition, *adnp* gene disruption in zebrafish leads to defective neurogenesis and reduced Wnt signaling. Our work provides important insights into the role of ADNP in neural development and the pathology of the Helsmoortel-Van der Aa syndrome caused by *ADNP* gene mutation.

## Abstract (web summary)

ADNP has been connected to neural developmental disorders. Here, the authors uncover a role for ADNP in neural induction and differentiation via β-Catenin stabilization, with ADNP disruption in zebrafish leading to defective neurogenesis and decreased Wnt signaling.

## Introduction

ADNP was first described as a neural protective protein and has been implicated in various neural developmental disorders and cancers^1^. This protein contains nine zinc fingers and a homeobox domain, suggesting that it functions as a transcription factor. The exact roles of ADNP remain unclear, but an increasing number of studies have shown that it may function as a key chromatin regulator by interacting with chromatin remodelers or regulators^2–6^.

Mouse *Adnp* mRNA is abundantly expressed during early gestation and reaches its maximum expression on E9.5. *Adnp−/−* mice are early embryonic lethal, displaying severe defects in neural tube closure and brain formation^7^. Of note, E8.5 *Adnp* mutant embryos exhibit ectopic expression of pluripotency gene *Pou5f1* and reduced expression of neural developmental gene *Pax6* in the anterior neural plate. These observations strongly suggest that ADNP plays essential roles during neurogenesis of mouse embryos.

De novo mutations in *ADNP* gene have recently been linked to neural developmental disorders, including the Helsmoortel-Van der Aa syndrome^8^. Patients may display multiple symptoms, which share many features with another neural developmental disorder: autism spectrum disorder (ASD). In fact, *ADNP* is one of the most frequent ASD-associated genes as it is mutated in at least 0.17% of ASD cases^9^. Due to the significant relevance of *ADNP* to neural developmental disorders, it is of great interests and importance in the field to elucidate the pathogenic mechanism by *ADNP* gene mutation^1,10^.

In this work, we hypothesize that loss of ADNP leads to neural developmental defects. We make use of ES cell directional neural differentiation as a model system to investigate the role of ADNP in embryonic neural development. We show that ADNP is required for proper neural induction by modulating Wnt/β-catenin signaling. Mechanistically, ADNP functions to stabilize β-Catenin through binding to its armadillo domain which prevents its interaction with key components of degradation complex: Axin and APC. Loss of ADNP leads to hyperphosphorylation of β-Catenin by GSK3β and subsequent degradation via ubiquitin-proteasome pathway, resulting in downregulation of neuroectoderm developmental genes. Small molecule-mediated activation of Wnt signaling can rescue the defects by loss of ADNP. This work provides important insights into the role of ADNP in neural development which would be useful for understanding the pathology of the Helsmoortel-Van der Aa syndrome caused by *ADNP* mutation.

## Results

### Generation and characterization of *Adnp−/−* ESCs

**Fig. 1:**
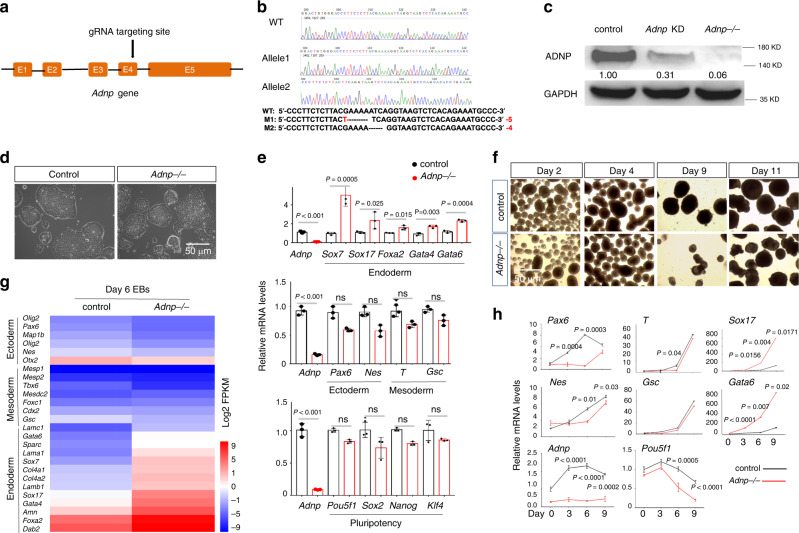
Generation of *Adnp−/−* ESCs. **a** Cartoon depicting the gRNA target sites at exon 4 of mouse *Adnp* gene. **b** Genotyping showing the mutant alleles. **c** Western blot analysis of ADNP levels in control, shRNA knockdown, and *Adnp−/−* ESCs. **d** Representative image showing morphology of control and early passaged *Adnp−/−* ESCs. **e** The mRNA expression of representative pluripotency-related, mesodermal, neuroectodermal, endodermal genes in control, and early passaged *Adnp−/−* ESCs (*n* = 3 per group). **f** Representative image showing morphology of embryoid bodies (EBs) at indicated time points. **g** Heat-map analysis of DEGs of the indicated lineage-specific genes for control and *Adnp−/−* ESC-derived day 6 EBs, based on three RNA-seq replicates. **h** qPCR analysis showing the dynamic expression of the indicated genes during EB formation of control and *Adnp−/−* ESCs. qRT-PCR was based on three biologically independent experiments (*n* = 3 per group). Data are presented as mean values ± SEM in (**e**, **h**). *p* values by two-tailed unpaired *t*-test are shown in (**e**, **h**). ns: not significant. Source data are provided as a Source Data file. Experiments were repeated at least two times in (**c**, **d**, **f**), and similar results were obtained.

To understand the molecular function of ADNP, we generated *Adnp* mutant ESCs by using CRISPR/Cas9 technology. Guide RNAs (gRNAs) were designed to target the 3′ end of exon 4 of the mouse *Adnp* gene (Fig. 1a). We have successfully generated two *Adnp* mutant alleles (4 and 5 bp deletion in exon 4 of the *Adnp* gene), as revealed by DNA genotyping around the CRISPR targeting site (Fig. 1b). ADNP protein was hardly detectable in *Adnp−/−* ESCs by western blot using ADNP antibodies from different resources, which strongly indicated that the mutant alleles are functional nulls (Fig. 1c).

In the traditional self-renewal medium containing LIF-KSR plus fetal bovine serum (FBS), the newly established *Adnp−/−* ESC colonies overall exhibited typical ESC-like morphology (Fig. 1d). To understand how loss of ADNP affected the global gene expression, we performed RNA-sequencing (RNA-seq) experiments for control and early passaged *Adnp−/−* ESCs. A total of 1,026 differentially expressed genes (DEGs) (log2 fold change > 1 and *p* < 0.05) were identified. Of which, an average of 766 genes were upregulated and 260 genes were downregulated (Supplementary Fig. 1a). RNA-seq analysis showed that the primitive endoderm genes such as *Gata6*, *Gata4*, and *Sox17* were slightly upregulated in the absence of ADNP. However, loss of ADNP effects on pluripotency-related, mesodermal, and neuroectodermal genes were subtle. Our qRT-PCR and immunofluorescence (IF) analyses confirmed the RNA-seq results (Fig. 1e; Supplementary Fig. 1b). Consistently, *Adnp−/−* ESCs can maintain self-renewal capacity for many generations before eventually adopting a flatten morphology and exhibiting reduced alkaline phosphatase activity, which could be rescued by reintroducing a FLAG-tagged ADNP into the mutant ESCs (Supplementary Fig. 1c).

To confirm that the observed phenotypes were not due to the possible off-target effect of the CRISPR/Cas9 technology, we also reduced *Adnp* transcript levels in ESCs by shRNA knockdown approach. The infection of lentivirus made from each of the two individual shRNAs led to about 70% reduction of *Adnp* mRNA levels (Fig. 1c; Supplementary Fig. 1d). *Adnp* knockdown ESCs morphologically resembled *Adnp−/−* ESCs (Supplementary Fig. 1e). qRT-PCR analysis of the selected genes revealed that the expression change of pluripotency and lineage specifying genes in *Adnp* knockdown ESCs was similar to that of *Adnp−/−* ESCs (Supplementary Fig. 1f).

### ADNP promotes ES cell directional neural differentiation

Next, we examined the pluripotency of *Adnp−/−* ESCs by performing the classical embryoid bodies (EBs) formation assay. Control ESCs were capable of forming large smooth spheroid EBs, whereas *Adnp−/−* ESCs formed smaller rough disorganized structures (Fig. 1f). To understand how loss of ADNP affected the global gene expression, we performed RNA-seq using day 6 EBs derived from control and *Adnp−/−* ESCs. A total of 2004 DEGs (log2 fold change > 1 and *p* < 0.05) were identified. Of which, an average of 1088 genes were upregulated and 916 genes were downregulated. Extra-embryonic endodermal genes such as *Gata6* and *Gata4* were substantially upregulated, while pluripotency genes such as *Nanog* and *Sox2*, and neural-ectodermal genes such as *Pax6, Olig2, Sox1, Nestin*, and *Otx2* were significantly downregulated (Fig. 1g). Our qRT-PCR analysis confirmed the RNA-seq results (Fig. 1h). Thus, based on the ESC-derived EB model, ADNP is required for the proper expression of neuroectodermal genes which was in line with the recent report^5^.

**Fig. 2:**
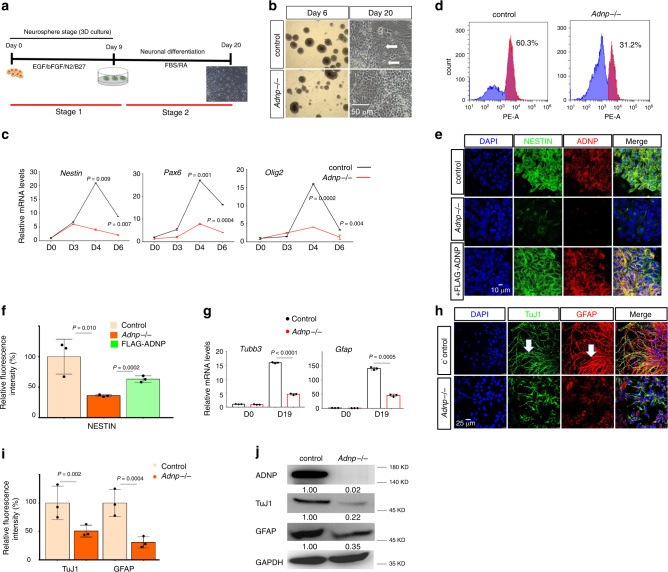
ADNP is required for proper ESC neural differentiation. **a** Cartoon showing the two-Stage ESC neural differentiation protocol. **b** Representative image showing morphology of day 6 and day 20 control and *Adnp−/−* ESC-derived neurospheres and neuronal cultures from three independent experiments with similar results. The white arrows pointing to the fiber-like neuronal structures. **c** The dynamic expression profile of representative neuroectodermal genes during control and *Adnp−/−* ESC neural induction. **d** Flow cytometry analysis for quantification of PAX6^+^ cells. Blue: isotype control; purple: experimental group using PAX6 antibody. **e** IF staining of neural progenitor marker NESTIN and ADNP for control, *Adnp−/−* ESC and FLAG-ADNP restoring *Adnp−/−* ESC-derived day 6 NPCs. Representative image from 3 to 5 random microscopic fields of three independent experiments with similar results. **f** Quantification of mean fluorescence intensity of NESTIN staining using ImageJ software. FLAG-ADNP rescued the fluorescence intensity of NESTIN staining in *Adnp−/−* ESC-derived neurospheres. **g** qRT-PCR analysis for *Tubb3* (encoding TuJ1) and *Gfap* for day 19 control and *Adnp−/−* ESC-derived neuronal cell cultures (*n* = 3 per group). **h** IF staining of neuronal marker TuJ1 and glial marker GFAP for day 19 control and *Adnp−/−* ESC-derived neuronal cell cultures. The white arrows showing the neuronal fiber structures. **i** Quantification of mean fluorescence intensity of TuJ1 and GFAP staining using ImageJ for panel (**h**). **j** WB analysis of TuJ1 and GFAP levels in day 19 control and *Adnp−/−* ESC-derived neuronal cell types. WB were repeated at least two times, and shown were the representative data. qRT-PCR was based on three biologically independent experiments in (**c**, **g**). Mean fluorescence intensity was calculated based on three biologically independent experiments (*n* = 3–5 different regions of interest per group) in (**f**, **i**). Data are presented as mean values ± SEM and *p* values by two-tailed unpaired *t*-test are shown in (**c**, **f**, **g**, **i**).

To better understand the role of ADNP in ESC neural differentiation, we directly differentiated control and *Adnp−/−* ESCs toward a neural cell fate. We adapted a modified three-dimensional (3D) floating neurosphere protocol that allows fast and efficient differentiation of ESCs into neural progenitors (Stage 1) and mature neurons (Stage 2) (Fig. 2a)^11–13^. At the end of Stage 1 of ESC neural differentiation, neurospheres from control ESCs were large, round, and even (Fig. 2b). The qRT-PCR results showed that day 6 control ESC-derived neurospheres abundantly expressed neural progenitor marker genes such as *Olig2*, *Pax2*, *Pax6*, and *Nestin* (Fig. 2c; Supplementary Fig. 2a). Flow cytometric assay revealed that more than 60% of neural progenitor cells (NPCs) were PAX6^+^, suggesting that our ESC neural induction was of high efficiency (Fig. 2d). In contrast, neurospheres from *Adnp−/−* ESCs were smaller, rough, and disorganized. The expression of neural progenitor markers was substantially reduced (Fig. 2c; Supplementary Fig. 2a, b). Flow cytometry results showed that only 31% of *Adnp−/−* ESC-derived NPCs were PAX6^+^ (Fig. 2d; Supplementary Fig. 2c). IF staining with NESTIN antibodies showed that NESTIN signals were greatly reduced in *Adnp−/−* ESC-derived neurospheres, which could be largely rescued by restoring FLAG-ADNP (Fig. 2e). Quantitative analysis showed that the mean fluorescence intensity of NESTIN staining was significantly lower in *Adnp−/−* ESC-derived neurospheres than in control counterparts (Fig. 2f).

When day 9 neurospheres from control ESCs were plated onto gelatin-coated plates to allow further differentiation for an additional 7–14 days (Stage 2), obvious neuronal fiber-like structures characterized by the branched and elongated morphology were observed (Fig. 2b). qRT-PCR analysis showed that day 19 ESC-derived cells abundantly expressed neuronal and glial marker genes *Tubb3* and *Gfap*, suggesting that more mature neural cell types were formed (Fig. 2g). IF staining results showed that TuJ1 and GFAP were abundantly expressed (Fig. 2i). In contrast, when neurospheres from *Adnp−/−* ESCs were plated onto gelatin-coated plates for further differentiation, few neuronal fibers were observed at day 19 (Fig. 2b). IF staining results showed that TuJ1 and GFAP signals were greatly reduced in *Adnp−/−* ESC-derived neuronal cells (~1/2 and 1/3 that of the control, respectively) (Fig. 2h, i). Quantitative analysis of TuJ1 and GFAP double staining revealed that there was a 60% reduction in mean fluorescence intensity in *Adnp−/−* ESC-derived neuronal cells compared with the control (Supplementary Fig. 2d). The qRT-PCR and WB results were consistent with that the expression of GFAP and TuJ1 was reduced in *Adnp−/−* ESC-derived neural cell cultures (Fig. 2g, j).

Taken together, we concluded that ADNP is required to promote ESC neural differentiation, and its loss leads to defective formation of NPCs and mature neuronal cell types.

### Loss of ADNP inhibits early neural developmental genes

During ES cell neural differentiation, ADNP levels were gradually induced, reaching its maximum level of expression at around day 4 (Supplementary Fig. 3a). The dynamic expression pattern of *Adnp* was closely correlated to that of the key neuroectoderm developmental genes such as *Olig2*, *Pax6,* and *Nestin* (Supplementary Fig. 3b). Importantly, loss of ADNP caused a significant downregulation of these genes (Fig. 2c). These observations implied that ADNP may function at early stage of neural differentiation program by controlling the expression of neuroectoderm developmental genes.

**Fig. 3:**
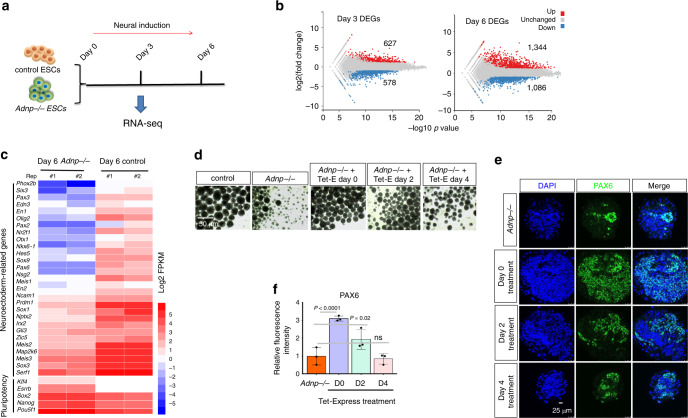
ADNP promotes the expression of neuroectoderm developmental genes. **a** Schematic representation of the design for RNA-seq experiments; **b** plot showing DEGs from day 3 and day 6 control and *Adnp−/−* ESC-derived neurospheres; RNA-seq for each time points were repeated at least two times. DEGs were defined by FDR < 0.05 and a Log2 fold change > 1. The numbers of upregulated and downregulated genes were shown as average. **c** Heat map illustrating the expression of selected neuroectoderm and pluripotency-related genes that were shown as log2 FPKM in day 6 control and *Adnp−/−* ESC-derived neurospheres. Each lane corresponds to an independent biological RNA-seq sample. **d** Representative morphology of day 6 *Adnp−/−* ESC-derived neurospheres after addition of Tet-Express proteins from the indicated time points. Tet stands for Tet-Express. Experiments were repeated two times, and similar results were obtained. **e** IF staining of PAX6 for day 6 *Adnp−/−* ESC-derived neurospheres after addition of Tet-Express proteins at the indicated time points. Experiments were repeated three times, and similar results were obtained. **f** Quantification of mean fluorescence intensity of PAX6 staining using ImageJ for panel (**e**), based on three biologically independent experiments (*n* = 3–5 different regions of interest per group). Data are presented as mean values ± SEM and *p* values by two-tailed unpaired *t*-test are shown. ns not significant. Source data are provided as a Source Data file.

To gain insights into the ADNP-dependent transcriptional program during ES cell differentiation toward a neural cell fate, we monitored the dynamic changes of gene expression by performing RNA-seq experiments at day 0, day 3/4, and day 6 of neural differentiation from control and *Adnp−/−* ESCs (Fig. 3a). In day 3 ESC-derived neurospheres, ~1200 DEGs (log2 fold change > 1 and *p* < 0.05) were identified, of which 627 genes were upregulated and 578 genes were downregulated. Among the downregulated genes were enriched for neuroectodermal markers such as *Sox3*, *Fgf5*, *Foxd3*, *Nestin*, *Sall2,* and *Nptx2*, while the upregulated genes were enriched for pluripotency-related markers and primitive endodermal markers. In day 6 ESC-derived NPCs, ~2430 DEGs (log2 fold change > 1 and *p* < 0.05) were identified, of which 1344 genes were significantly upregulated and 1086 genes were downregulated (Fig. 3b). Of note, genes implicated in neuroectoderm development such as *Fgf5*, *Hes5*, *Musashi1*, *Sox1*, *Sox3*, *Pax2/6*, *Pax3/7*, *Foxd3*, *Nestin*, *Irx2*, *Cdx2*, *Sall2*, and *Nptx2* were significantly downregulated. Heat-map analysis of day 3 and day 6 DEGs showed that there was a clearly decreased expression of neuroectoderm-related genes in the absence of ADNP (Fig. 3c; Supplementary Fig. 3c). The qRT-PCR analysis of the selected genes confirmed the RNA-seq results (Fig. 2c).

If ADNP functions upstream of neuroectoderm developmental genes, restoring ADNP should rescue the neural differentiation defects from *Adnp−/−* ESCs. To this end, we took use of a Tet-Express inducible transgenic *Adnp−/−* ES cell line in which 3×FLAG-ADNP can be induced by the addition of the Tet-Express protein (Supplementary Fig. 3d). We found that the addition of Tet-Express at early time points (day 0 or 2) could partially rescue the morphological and gene expression defects, while addition of Tet-Express after day 4 had minimal effects (Fig. 3d–f; Supplementary Fig. 3e).

Together, these data strongly suggested that ADNP performs an important role at the early stage of neural differentiation by promoting the expression of neuroectoderm developmental genes.

### Wnt/β-catenin signaling is reduced in the absence of ADNP

**Fig. 4:**
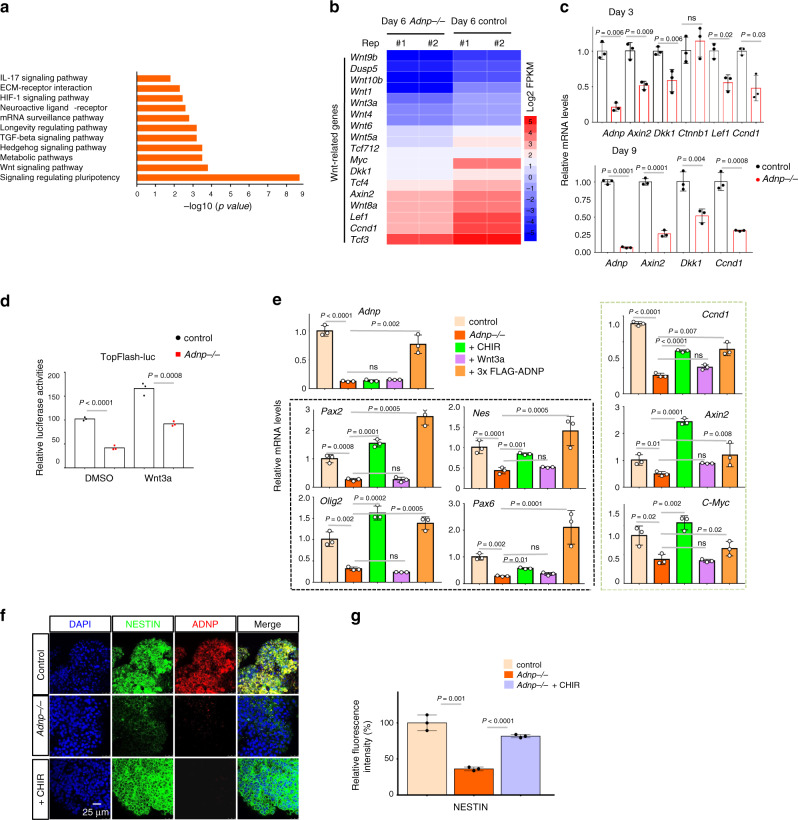
Wnt signaling is impaired in the absence of ADNP. **a** Dissection of KEGG data of DEGs from day 3 and day 6 control and *Adnp−/−* ESC-derived neurospheres, showing enrichment of the Wnt signaling pathway. Results are shown as −log10 (*p* value). **b** Heat map illustrating the expression of selected Wnt-related genes that were shown as log2 FPKM in day 6 control and *Adnp−/−* ESC-derived neurospheres. Each lane corresponds to an independent biological RNA-seq sample. **c** qRT-PCR analysis for the indicated Wnt target genes for day 3 and day 9 control and *Adnp−/−* ESC-derived neurospheres. qRT-PCR was based on three biologically independent experiments (*n* = 3 per group). **d** TopFlash luciferase activity assay for lysates from day 3 control and *Adnp−/−* ESC-derived neurospheres, in the absence or presence of Wnt3a. Data are based on two biologically independent experiments, and similar results were obtained. **e** Rescue of the expression of the indicated neural developmental genes and putative Wnt target genes by addition of CHIR and Wnt3a, or by restoring 3×FLAG-ADNP. Data are based on three biologically independent experiments, and similar results were obtained. Genes in black dashed box are representative neurodevelopmental genes, and genes in green dashed box are representative Wnt target genes. **f** Rescue of NESTIN expression by addition of CHIR. Shown is the representative IF staining of NESTIN for day 6 control and *Adnp−/−* ESC-derived neurospheres (*n* = 3 per group). **g** Quantification of mean fluorescence intensity of NESTIN staining using ImageJ for panel (**f**) based on three biologically independent experiments (*n* = 3–5 different regions of interest per group). Data are presented as mean values ± SEM, and *p* values by two-tailed unpaired *t*-test are shown in (**c**, **d**, **e**, **g**). Source data are provided as a Source Data file for (**c**, **d**, **e**).

Next, we sought to explore the underlying mechanisms by which ADNP controls ES cell neural induction. To answer the question, we performed KEGG pathway enrichment analysis of the DEGs identified in our RNA-seq results. This revealed a strong enrichment among DEGs for annotations associated with signal transduction as well as signaling molecules and interaction (Supplementary Fig. 4a). Detailed analysis showed that ADNP-dependent downregulated genes were highly enriched for gene networks regulating Wnt signaling (Fig. 4a). Consistently, the heat-map analysis revealed that there was a clear reduction in transcript levels of Wnt-related genes (Fig. 4b; Supplementary Fig. 4b). This observation strongly suggested that loss of ADNP blocks Wnt signaling pathway. To confirm the RNA-seq results, qRT-PCR for day 3 and day 9 ESC-derived neurospheres was performed to analyze the expression of Wnt target genes such as *Ccnd1, Axin2, Lef1*, and *Tcf712*. The expression of these genes at both time points was significantly reduced in the absence of ADNP (Fig. 4c). Note that the expression of *Ctnnb1* gene, which encodes for β-catenin, was barely changed. To further confirm that Wnt signaling was compromised in the absence of ADNP, we performed TopFlash luciferase reporter assay for day 3 control and mutant ESC-derived NPCs. As shown in Fig. 4d, loss of ADNP substantially decreased the TopFlash reporter activities, in the presence and absence of Wnt3a.

On the other hand, we investigated whether overexpressing ADNP could facilitate Wnt signaling. To this end, we performed the TopFlash reporter assay in HEK293T cells transfected with an increasing amount of plasmids encoding ADNP. As shown in Supplementary Fig. 4c, the TopFlash reporter activities were enhanced in a ADNP dose-dependent manner, in the presence and absence of Wnt3a. Based on these data, we concluded that ADNP positively regulates Wnt/β-catenin signaling.

The above data suggested that loss of ADNP function may affect neural induction by inhibiting Wnt signaling. If this is the case, enhancing Wnt signaling should rescue the neural defects from *Adnp−/−* ESCs. CHIR99021 (CHIR) is a well-characterized canonical Wnt signaling pathway activator. We found that addition of 3 μM CHIR partially rescued the gene and protein expression as well as morphological defects of day 6 *Adnp−/−* ESC-derived NPCs (Fig. 4e, f; Supplementary Fig. 4d, e). Quantitative analysis showed that the fluorescence intensity of NESTIN staining was significantly higher in cells treated with CHIR than in cells without CHIR treatment (Fig. 4g). However, treatment with different doses of Wnt3a proteins had little effect (Fig. 4e; Supplementary Fig. 4f, g). These data suggested that ADNP may regulate Wnt signaling downstream of the activity of Wnt ligands but upstream or parallel to GSK3β^14,15^.

Taken together, we concluded that ADNP is critical for ES cell neural differentiation by promoting the Wnt/β-catenin signaling pathway. In the absence of ADNP, Wnt signaling is impaired and the ability of ESC differentiation toward neuroectodermal lineage cell fate is compromised.

### Identifying β-catenin as an ADNP interacting protein

**Fig. 5:**
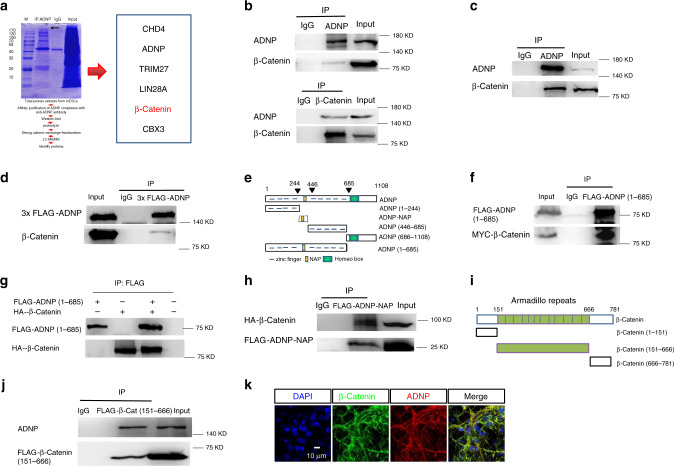
Identifying β-catenin as an ADNP interacting protein. **a** Schematic representation showing the experimental design of IP in combination mass spectrometry assay (left) and a list of representative ADNP interacting proteins (right). **b** Co-IP data for endogenous ADNP and β-catenin in ESCs. Up: IP ADNP followed by WB β-catenin; bottom: IP β-catenin followed by WB ADNP. **c** Co-IP data for endogenous ADNP and β-catenin of day 3 ESC-derived neurospheres. **d** Co-IP data for FLAG-ADNP and β-catenin in 3×FLAG-ADNP overexpressing *Adnp−/−* ESCs. **e** Schematic representation of the full-length and the truncated ADNP mutants. **f** IP of FLAG-ADNP-Nter (1–685) and Myc-β-catenin in HEK293T cells. **g** IP of in vitro synthesized HA-β-catenin and FLAG-ADNP. **h** Co-IP of FLAG-ADNP-NAP and HA-β-catenin in HEK293T cells. **i** Schematic representation of the full-length and the truncated β-catenin mutants. **j** Co-IP of FLAG-β-catenin (151–666) and ADNP in HEK293T cells. **k** Co-localization of ADNP and β-catenin in day 19 ESC-derived neuronal cell cultures. All WB and IF experiments were repeated at least two times. Similar results were obtained and shown are the representative images.

In our immunoprecipitation combined with mass spectrometry assay (IP-Mass Spec), β-Catenin was detected as one of the top hits of ADNP immunoprecipitates (Fig. 5a). As β-Catenin is the key player in the canonical Wnt signaling pathway, we hypothesized that ADNP may regulate Wnt signaling by targeting β-Catenin. We first investigated whether ADNP interacts with β-catenin by performing co-immunoprecipitation (Co-IP) experiments. The Co-IP results showed that endogenous ADNP interacts with β-Catenin in both ESCs and day 3 ESC-derived NPCs, albeit exhibiting much stronger interaction in NPCs than in ESCs (Fig. 5b, c). We also performed the Co-IP experiments using the 3×FLAG-ADNP overexpressing *Adnp−/−* ESC line. FLAG-ADNP readily pulled down endogenous β-Catenin in day 3 ESC-derived NPCs (Fig. 5d).

Next, we mapped the interacting domains for ADNP and β-catenin by co-transfecting plasmids encoding the truncated forms of ADNP and β-catenin into HEK293T cells (Fig. 5e). We found that it was the N-terminal fragment (1–685) but not the C-terminal fragment of ADNP (686–1108) that is responsible to interact with β-catenin (Fig. 5f; Supplementary Fig. 5a). To investigate whether they interact directly, we used a reticulate system to synthesize FLAG-ADNP (1–685) and HA-β-catenin. When they were mixed together, FLAG-ADNP (1–685) could readily pull down HA-β-catenin (Fig. 5g). Besides the zinc finger motifs, ADNP-Nter (1–685) contains NAP (also called davunetide or CP201), an 8-amino acid neuroprotective peptide (354–361 within the ADNP-Nter) derived from the ADNP protein^16–19^. NAP was shown to enhance microtubule assembly and neurite outgrowth and protect neurons and glia exposed to toxins in mouse and rat models^16^. A recent study has shown that major symptoms of the Helsmoortel-Van der Aa syndrome could be partially ameliorated by daily NAP treatment^20^. We wondered whether NAP alone could be sufficient to interact with β-catenin. When plasmids encoding FLAG-ADNP-NAP and HA-β-catenin were co-transfected into HEK293T cells, FLAG antibodies could pull down HA-β-catenin (Fig. 5h). Of note, ADNP-Nter without NAP could still interact with β-catenin but this interaction was much weaker than that of ADNP-Nter and β-catenin (Supplementary Fig. 5b).

Next, we determined the fragments of β-catenin that are responsible for interaction with ADNP. β-catenin has an N-terminal domain (residues 1–150) that harbors the binding site for GSK3β and CK1 phosphorylation, a central armadillo domain (residues 151–666) composed of 12 armadillo repeats, and a C-terminal domain (residues 667–781)^15^ (Fig. 5i). Our mapping experiments showed that the central armadillo domain of β-catenin strongly interacts with ADNP, while the N-terminal (1–150) and the C-terminal fragment (667–781) barely interact with ADNP (Fig. 5j; Supplementary Fig. 5c). These data indicated that the armadillo domain of β-catenin primarily mediates the interaction with ADNP.

Finally, IF staining was performed to investigate the cellular co-localization of ADNP and β-catenin. During ES cell neuronal differentiation, there was a marked change in the intra-cellular localization of ADNP proteins: from predominantly nuclear localization in undifferentiated ESCs to predominantly cytoplasmic localization in ESC-derived NPCs and mature neurons (Supplementary Fig. 5d; Fig. 5k). This result was analogous to the previous report using the pluripotent P19 cells^21^.

### ADNP stabilizes β-catenin during ESC neural differentiation

**Fig. 6:**
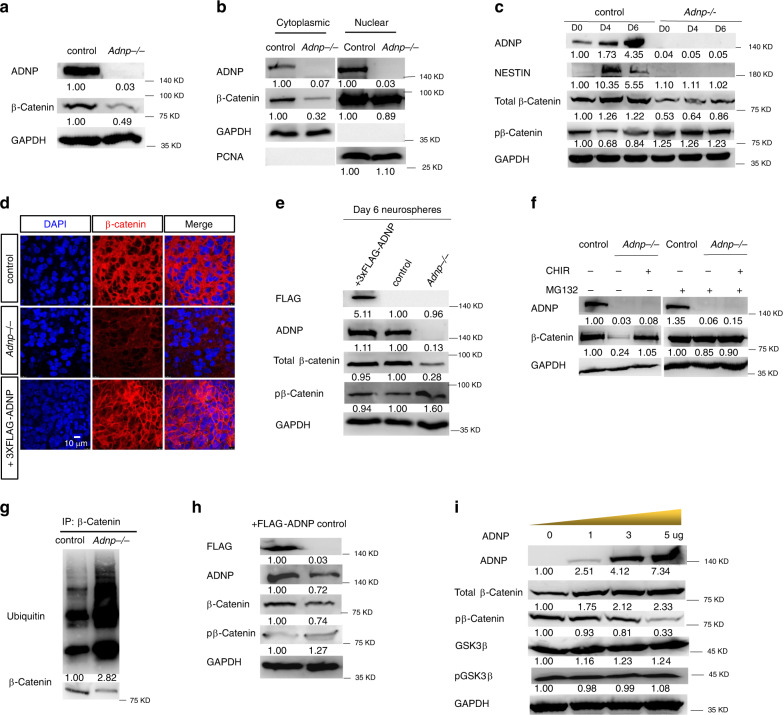
ADNP stabilizes β-catenin during ESC neural differentiation. **a** WB showing total β-catenin levels in day 3 control and *Adnp−/−* ESC-derived neurospheres. **b** WB showing β-catenin levels in the cytoplasmic and the nuclear fraction of lysates from day 3 control and *Adnp−/−* ESC-derived neurospheres. Note that β-catenin levels were significantly reduced in the cytoplasmic but slightly reduced in the nuclear fraction of lysates. **c** Time course WB assay showing total and phosphorylated β-catenin levels during control and mutant ESC differentiation toward a neural fate. **d** Representative image of IF staining of β-catenin in day 19 ESC-derived neuronal cultures; **e** restoring FLAG-ADNP in mutant ESCs could rescue the β-catenin levels in day 6 neurospheres. **f** Representative WB showing β-catenin levels in the combination treatment of CHIR and MG132. **g** Representative WB showing the ubiquitylation levels of β-catenin in control and *Adnp−/−* ESCs. **h** Representative WB showing that total β-catenin levels were slightly elevated and p-β-catenin levels were slightly reduced in day 3 neurospheres from FLAG-ADNP overexpressing ESCs. **i** Representative WB showing the expression of the indicated proteins in HEK293T cells that were transfected with an increasing dose of ADNP. WB and IF staining experiments have been repeated at least two times and similar results were obtained.

Given the physical association of ADNP and β-Catenin, we asked whether ADNP is involved in the turnover of β-catenin protein. First, we examined the expression levels of β-catenin in day 3 control and *Adnp−/−* ESC-derived neurospheres. Loss of ADNP led to a significant reduction of total β-catenin levels (Fig. 6a). This reduction was primarily contributed by the cytoplasmic but not the nuclear fraction of β-catenin (Fig. 6b). Throughout ESC neural differentiation, total β-catenin levels remained lower in the absence of ADNP than in its presence (Fig. 6c, d; Supplementary Fig. 6a, b). Importantly, β-catenin levels in day 6 *Adnp−/−* ESC-derived neurospheres could be largely rescued by restoring FLAG-ADNP (Fig. 6e). When ESC-derived NPCs were treated with the proteasome inhibitor MG132, loss of ADNP failed to induce β-catenin degradation, suggesting that ADNP-regulated β-catenin degradation might be dependent on the ubiquitin-proteasome pathway (Fig. 6f). Further studies showed that loss of ADNP strongly enhanced the ubiquitylation levels of β-catenin, which could be reversed by restoring 3×FLAG-ADNP (Fig. 6g; Supplementary Fig. 6c).

On the other hand, we investigated the effect of overexpressing ADNP on total β-catenin levels. Total β-catenin levels were slightly elevated in day 3 neurospheres from FLAG-ADNP overexpressing ESCs compared with the control counterpart (Fig. 6h). Overexpressing ADNP also led to higher expression of β-catenin in HEK293T cells (Fig. 6i). Next, we asked whether overexpression of ADNP proteins affects the stability of co-expressed β-catenin by co-transfecting plasmids encoding FLAG-ADNP and HA-β-catenin into 293 T cells. The IP results showed that FLAG-ADNP could stabilize HA-β-catenin in a dose-dependent fashion (Supplementary Fig. 6d). Combining both loss of function and gain of function data, we concluded that ADNP is a positive regulator of β-catenin stabilization during ESC differentiation toward a neural cell fate.

A previous study has suggested that ADNP may negatively regulate the phosphorylation activity of GSK3β^10,22^. To investigate whether ADNP regulates the stability of β-catenin through modulating GSK3β activities, WB analysis for phospho-GSK3β (Ser9) and total GSK3β was performed for day 3 control and *Adnp−/−* ESC-derived neurospheres. We found that both the phospho-GSK3β and total GSK3β levels were barely altered in the absence of ADNP (Supplementary Fig. 6e). In addition, overexpression of ADNP had little effect on phospho-GSK3β and total GSK3β levels (Fig. 6i). These results suggested that ADNP may not modulate the levels or activities of GSK3β.

**Fig. 7:**
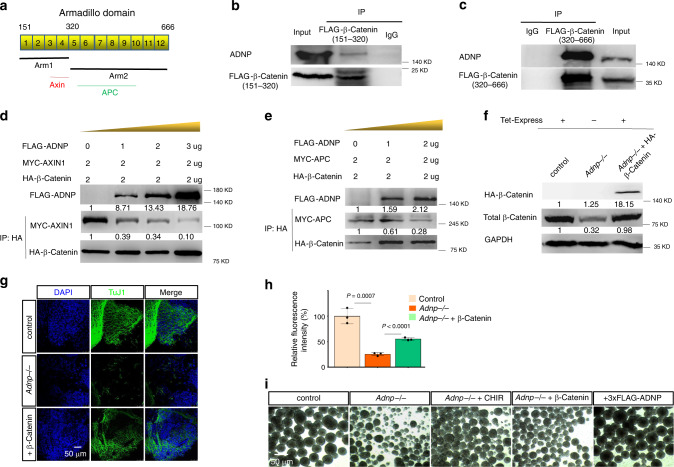
ADNP stabilizes β-catenin by preventing the formation of degradation complex. **a** Cartoon showing the armadillo domain of β-catenin. Arm1 contains armadillo repeats 1–4 and Arm2 contains repeats 5–9. Known core binding armadillo repeats of β-catenin for Axin and APC interaction were indicated with red and green bars, respectively. **b** IP experiment showing that the Arm1 fragment can interact with ADNP in HEK293T cells. **c** IP experiment showing that the Arm2 fragment can interact with ADNP in HEK293T cells. **d** The relationship between ADNP–β-catenin and β-catenin–Axin interactions was examined by performing competitive protein-binding assay. An increasing dose of plasmids encoding FLAG-ADNP and plasmids encoding MYC-AXIN1 and HA-β-catenin was co-transfected into HEK293T cells. Lysates were extracted from the transfected cells and pull-downed by HA antibody followed by WB using MYC and HA antibodies. WB showing that the amount of Axin co-immunoprecipitated with β-catenin became reduced by the increased addition of ADNP. **e** The competitive protein-binding assay showing that the amount of APC co-immunoprecipitated with β-catenin became reduced by the increased addition of ADNP. **f** WB showing the expression levels of HA-β-catenin by the addition of Tet-Express protein in the inducible HA-β-catenin *Adnp−/−* ESCs. **g** IF staining showing the rescue of TuJ1 levels in day 19 *Adnp−/−* ESC-derived neuronal cell cultures by adding Tet-Express transactivator daily at early stage of neural induction. **h** Quantification of mean fluorescence intensity of TuJ1 staining using ImageJ for panel (**g**), based on three biologically independent experiments (*n* = 3–5 different regions of interest per group). Data are presented as mean values ± SEM and *p* values by two-tailed unpaired *t*-test are shown. **i** The representative morphology of day 6 neurospheres derived from control, *Adnp−/−* ESCs, *Adnp−/−* ESCs treated with CHIR, as well as FLAG-ADNP and HA-β-catenin restoring *Adnp−/−* ESCs, and the rescue experiments were repeated two times. The WB and IP experiments have been repeated two times and similar results were obtained.

Next, we asked whether ADNP is involved in the regulation of β-catenin phosphorylation by GSK3β. We found that this was the case because phospho-β-catenin levels were elevated in the absence of ADNP and were reduced when ADNP was overexpressed (Fig. 6c–i). To understand the molecular mechanism by which ADNP may regulate β-catenin phosphorylation levels, we further mapped the armadillo repeat domain that is responsible for interacting with ADNP. The armadillo repeat domain of β-catenin has 12 armadillo repeats, containing overlapping binding sites for many binding partners, including Axin and APC^15^. It is well-known that Axin, APC, and β-catenin form the degradation complex that promotes the GSK3β-mediated phosphorylation and subsequent degradation of β-catenin. We divided the armadillo repeat domain into two parts: one contains repeats 1–4 (Arm1) which mediates β-catenin for the interaction with the scaffolding protein Axin, and another one composed of repeats 5–12 (Arm2) which interacts with APC (Fig. 7a)^23^. The IP results showed that both the Arm1 and Arm2 were able to interact with ADNP although Arm2 displayed a stronger capacity to associate with ADNP (Fig. 7b, c). Based on the results, we hypothesized that ADNP may protect β-catenin from hyperphosphorylation by GSK3β through competing with Axin and APC. To directly investigate the possibility, we examined the relationship between ADNP–β-catenin and β-catenin–Axin interactions by performing competitive protein-binding assay. As shown in Fig. 7d, the amount of Axin co-immunoprecipitated with β-catenin became reduced by the increased addition of ADNP. Similar result was obtained for the relationship between ADNP–β-catenin and β-catenin–APC interactions (Fig. 7e).

To further confirm that ADNP can protect β-catenin from hyperphosphorylation by GSK3β, we transfected an increasing dose of plasmids encoding wild-type ADNP or ADNP-Cter (686–1108) mutants into HEK293T cells. We found that overexpressing wild-type ADNP but not ADNP-Cter decreased the p-β-catenin levels and increased the total β-catenin levels, in a dose-dependent fashion (Fig. 6i). This data suggested that the interaction between ADNP and β-catenin is required for β-catenin stabilization through modulating its phosphorylation levels.

Finally, we asked whether increasing β-catenin protein levels could rescue the neural differentiation defects caused by loss of ADNP. To this end, we made a Tet-Express inducible HA-β-catenin transgenic *Adnp−/−* ESC line and used it for neural induction. Before performing rescue experiments, we confirmed that addition of Tet-Express proteins could induce β-catenin levels similar to that of control ESCs (Fig. 7f). The ability of *Adnp−/−* ESCs to differentiate into neural progenitors and neuronal cells was largely rescued by the addition of Tet-Express, revealed by the quantitative analysis of NESTIN and TuJ1 staining (Fig. 7g–i; Supplementary Fig. 7a, b). Thus, restoring β-catenin levels had similar rescue effects as CHIR99021 treatment or restoring ADNP on *Adnp−/−* ESC neural induction and differentiation.

### Loss of *adnp* leads to reduced Wnt signaling in zebrafish

Zebrafish has been proven to be a good in vivo model to study vertebrate neurogenesis and pathology of ASD-related genes^24,25^. Zebrafish has two *adnp* homologs: *adnpa* and *adnpb*, and the expression of which has been reported (Supplementary Fig. 8a)^26^. After 24 h, *adnpa* expression was restricted to the forebrain, the midbrain, the mid-hindbrain boundary and eye area (Supplementary Fig. 8b).

**Fig. 8:**
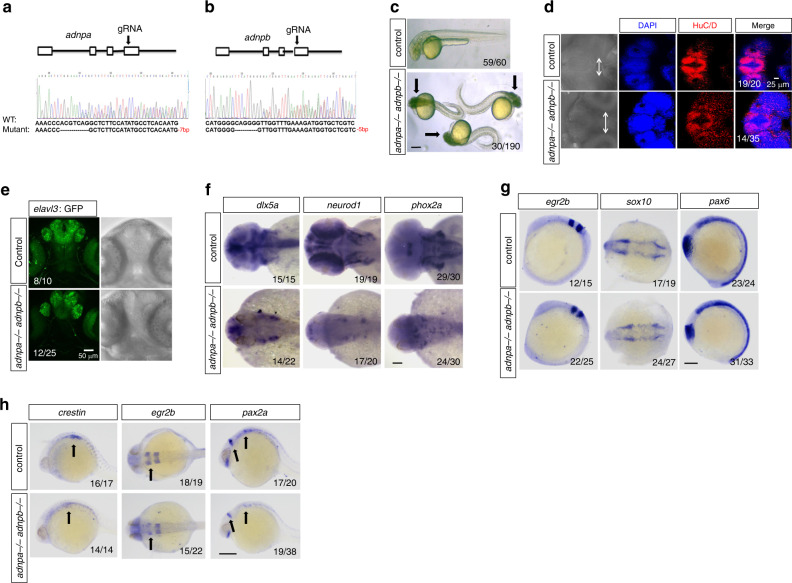
Loss of *adnp* leads to the defective neural development in zebrafish embryos. **a** CRISPR/Cas9 gRNA design and the genotyping result of the *adnpa* mutant allele. **b** gRNA design and the genotyping result of *adnpb* mutant allele; **c** representative morphology of 2 dpf control and *adnpa adnpa* double mutants. Black arrow showing the obvious cell death (black area) in head region of mutant embryos. **d** 3D Z-stack image showing the reduced expression of HuC/D in brain of 36 hpf control and double mutant zebrafish embryos. Arrows show the distance between the convex tip of the eye cups. **e** GFP fluorescence signal showing the reduced *elavl3*-GFP levels in *adnpa adnpb* deficient embryos. **f** Whole-mount in situ hybridization (WISH) images for the indicated neural markers for 2 dpf control and double mutant embryos. **g** WISH images for the indicated neural markers for 13 hpf control and double mutant embryos. **h** WISH images for the indicated neural markers for 24 hpf control and double mutant embryos. Black arrows pointing to where expression levels were reduced. All experiments were repeated at least two times, and similar results were obtained. Scale bars: 200 μm in (**c**), 100 μm in (**d**–**h**).

To understand the function of zebrafish Adnp (zAdnp), we generated *adnpa* and *adnpb* mutants using the CRISPR/Cas9 technology (Fig. 8a, b). Both *adnpa* and *adnpb* mutant embryos can grow up to adults without apparent morphological defects (Supplementary Fig. 8c). We went on to obtain *adnpa adnpb* double mutant lines by crossing *adnpa−/−* and *adnpb−/−* zebrafish. The majority of *adnpa adnpb* double mutant embryos appeared normal at 1 dpf, although a small portion of the embryos exhibited axis formation defects (Supplementary Fig. 8d). Strikingly, about 16% (30/190) of double mutant embryos at 36–48 hpf displayed abnormal body morphology with massive neuronal death in brain (Fig. 8c). To investigate whether there was neuronal death phenotype, IF assay was performed to detect the expression of HuC/D for 36 hpf control and mutant embryos. As shown in Fig. 8d, the HuC/D signal was decreased in double mutant embryos. To confirm the result, we crossed *adnpa−/− adnpb−/−* females to the transgenic Tg (*elavl3*: GFP) (formerly HuC: GFP) male fish^27^. We found that the GFP signal was significantly reduced in Adnp deficient embryos (Fig. 8e). Furthermore, whole-mount in situ hybridization (WISH) results showed that the expression of neural genes such as *dlx5a, neurod1*, and *phox2a* was greatly reduced in day 2 double mutant embryos compared with sibling controls (Fig. 8f).

To investigate whether there were early neurodevelopmental defects in *adnpa adnpb* double mutant embryos, we examined the expression of a set of early neural genes. The expression of early neural markers such as *foxd3, pax6, sox10*, and *egr2b* was comparable between control and mutant embryos at 8 and 13 hpf (Fig. 8g; Supplementary Fig. 8e). However, when 24 hpf embryos were analyzed, we found that there was a slightly reduced expression of neural markers such as *crestin*, *pax2* and *egr2b* (Fig. 8h). These data suggested that zAdnp might not be required for the initial specification of neural precursors but required for neural and neuronal differentiation at a slightly later developmental stage.

**Fig. 9:**
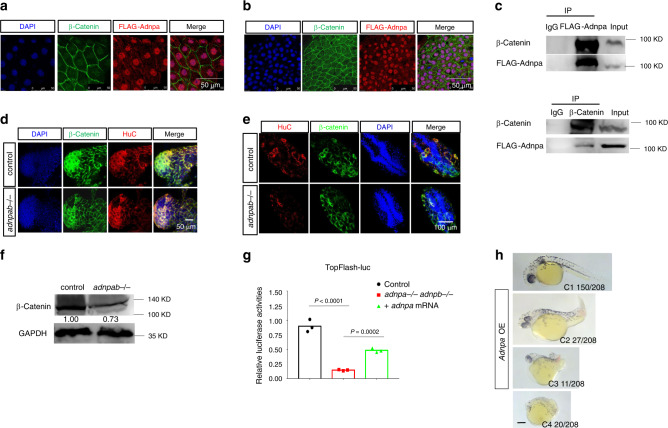
Loss of *adnp* leads to reduced β-catenin levels and Wnt signaling in zebrafish embryos. **a** IF staining of FLAG and β-catenin showing the co-localization of FLAG-Adnpa and β-catenin in 3.5 hpf *adnpa−/− adnpb−/−* embryos injected with mRNAs encoding FLAG-Adnpa. FLAG-Adnpa is predominantly expressed in the nucleus. **b** IF staining of FLAG and β-catenin showing the co-localization of FLAG-Adnpa and β-catenin in 6 hpf *adnpa−/− adnpb−/−* embryos injected with mRNAs encoding FLAG-Adnpa. FLAG-Adnpa is predominantly expressed in the nucleus. **c** Co-IP data showing FLAG-Adnpa interact with β-catenin in 24 hpf *adnpa−/− adnpb−/−* embryos injected with mRNAs encoding FLAG-Adnpa. **d** IF staining of β-catenin and HuC showing slightly reduced HuC and β-catenin levels in 16 hpf *adnpa−/− adnpb−/−* (*adnpab−/−*) embryos. **e** IF staining of β-catenin and HuC showing significantly reduced HuC and β-catenin levels in 24 hpf *adnpa−/− adnpb−/−* embryos. **f** WB data showing slightly reduced β-catenin levels in 24 hpf *adnpa−/− adnpb−/−* embryos. **g** TopFlash luciferase activity assay for total lysates made from 24 hpf control, double mutant embryos and double mutant embryos injected with *adnpa* mRNAs. Three pools of ten embryos each (*n* = 3 per group) were lysed with the passive lysis buffer and assayed for luciferase activity. Data are based on two biologically independent experiments, and similar results were obtained. **h** Representative morphology of 2 dpf *adnpa* overexpressing embryos. The dorsalized phenotypes (C1–C4) were according to the DV patterning index^38^. All experiments were repeated at least two times, and similar results were obtained. Shown are representative images. Scale bars: 50 μm in (**a**, **b**, **d**), 100 μm in (**e**), and 200 μm in (**h**).

Next, we asked whether zAdnp regulates zebrafish neurogenesis through Wnt signaling by targeting β-catenin. To this end, we first investigated whether zAdnp interacts with β-catenin in zebrafish embryos. mRNAs encoding FLAG-tagged Adnpa were injected into one-cell stage double mutant embryos, and IF assay was performed to detect FLAG-Adnpa in embryos at different developmental stages. The IF results showed that FLAG-Adnpa was ubiquitously expressed and predominantly localized in the nucleus of 3.5 and 6 hpf embryos, and predominantly localized in the cytoplasm of 13 and 24 hpf embryos (Fig. 9a, b; Supplementary Fig. 9a, b). To investigate the cytoplasmic localization of Adnpa in the context of embryonic neurogenesis, mRNAs encoding FLAG-tagged Adnpa were injected into one-cell stage Tg (*sox10*: GFP) or Tg (*elavl3*: GFP) zebrafish embryos. The IF results showed that all Sox10 or HuC positive cells were FLAG positive, which supported that Adnpa plays important role in zebrafish neurogenesis (Supplementary Fig. 9c, d). Double IF staining with FLAG and β-catenin antibodies showed that FLAG-Adnpa was extensively co-localized with endogenous β-catenin in the cytoplasm of embryos at 13 and 24 hpf. In pre-gastrulating and gastrulating embryos, FLAG-Adnpa and β-catenin were also co-localized predominantly in the nucleus (Fig. 9a, b). Thus, FLAG-Adnpa cellular localization was shifted from predominantly nuclear to cytoplasmic during early embryogenesis. Finally, the Co-IP results showed that FLAG-Adnpa interacts with β-catenin in zebrafish embryos (Fig. 9c).

Next, we asked whether loss of Adnp led to reduced β-catenin levels or alteration of β-catenin cellular localization. It is well-known that β-catenin nuclear localization at dorsal blastomere is critical for proper body axis formation of zebrafish embryos^28–30^. IF staining using β-catenin antibodies showed that neither β-catenin levels nor its cellular localization was significantly changed in double mutant embryos (Supplementary Fig. 9f, g). Double IF staining of β-catenin and HuC showed that β-catenin levels were slightly reduced in HuC^+^ cells in 16 hpf mutant embryos, and significantly reduced in 24 hpf mutant embryos (Fig. 9d, e). WB analysis showed that total β-catenin levels were slightly reduced in double mutant embryos (Fig. 9f). To investigate whether Wnt signaling was reduced in double mutant embryos, the TopFlash luciferase assay was performed. As shown in Fig. 9g, the TopFlash luciferase activities were significantly reduced in mutant embryos, which could be partially rescued by micro-injection of *adnpa* mRNAs.

On the other hand, we overexpressed Adnpa by micro-injecting *adnpa* mRNAs into one-cell stage wild-type embryos. Adnpa overexpressing embryos exhibited phenotypes ranging from weakly dorsalized to severely dorsalized (Fig. 9h), which resembled that of β-catenin overexpressing embryos^29^. Consistently, there was a slight expansion of dorsal marker *chd* and reduction of ventral marker *eve1*, and an increased expression of Wnt target gene *tbx6* in Adnpa overexpressing embryos (Supplementary Fig. 9h). To further confirm that overexpression of Adnpa caused dorsalization of embryos, we directly examined the dorsal organizer for shield-stage control and Adnpa overexpressing embryos. We found that overexpression of Adnpa led to an enlarged dorsal organizer (Supplementary Fig. 9i).

Taken together, we concluded that overexpression of zAdnp leads to dorsalized embryos similar to β-catenin overexpression, and loss of zAdnp leads to reduced Wnt/β-catenin signaling and defective neurogenesis in zebrafish embryos.

## Discussion

De novo mutations in *ADNP* have been recently linked to the Helsmoortel-Van der Aa syndrome. And *ADNP* has been proposed as one of the most frequent ASD-associated genes as it is mutated in at least 0.17% of ASD cases. It is therefore important to determine the molecular function of this protein, especially during embryonic neurogenesis.

We found that loss of ADNP had little effect on the mRNA levels of *Ctnnb1* gene which encodes β-catenin in ESCs. This rules out that ADNP controls β-catenin levels transcriptionally. During ESC differentiation toward a neural fate, the cellular localization of ADNP was shifted from predominantly nuclear to cytoplasmic. This nucleus-cytoplasm translocation during neural differentiation was also observed by Dr. Gozes’s group using P19 cell line^21^. These observations suggest that the intra-cellular localization of ADNP is tightly regulated during neural induction, and/or that ADNP plays an important role in the cytoplasm for the induction, maintenance and maturation of neural cell types. By IP-Mass Spec, β-catenin was identified as a top ADNP interacting protein. Considering that Wnt/β-catenin signaling pathway is critically required for neural induction and differentiation, we reasoned that ADNP may control neurogenesis by regulating Wnt signaling via targeting β-catenin. We provided solid evidences that ADNP promotes neural induction and neuronal differentiation by directly associates with β-catenin and regulates its stability.

Canonical Wnt signaling and β-catenin have been implicated in mouse and human embryonic stem cell maintenance^31–35^. In this work, *Adnp−/−* ESCs only displayed a slightly reduced β-catenin levels, and they remained undifferentiated for many generations in the traditional LIF-KSR medium. During ESC neural differentiation, however, a greater reduction of β-catenin levels was observed. This suggests that ADNP is not a core component of the Wnt signaling pathway in pluripotent ESCs. Instead, it may function as a temporal-specific regulator of Wnt signaling during neurogenesis, which is better supported by our zebrafish studies.

Using zebrafish as an in vivo model, we found that a small portion of *adnpa adnpb* double mutant embryos exhibit neurodevelopmental defects and massive neuronal cell death in the head region, which recapitulates the phenotype of *Adnp* deficient mouse embryos. zAdnpa is predominantly nuclear localized in pre-gastrulating and gastrulating embryos. Soon after gastrulation, zAdnpa is predominantly co-localized with β-catenin in the cytoplasm of HuC^+^ or Sox10^+^ neural cells. Based on the temporal change of intra-cellular localization of Adnpa, we propose that zAdnp functions to regulate β-catenin levels during zebrafish neurogenesis, which is analogous to our ESC work: ADNP stabilizes β-catenin primarily in the cytoplasm by association with it during ESC neural differentiation (but not in undifferentiated ESCs).

Taken together, we propose that ADNP is a temporal- or tissue-specific modulator of the Wnt signaling pathway which is required for neural induction and neuronal differentiation. This may explain why *Adnp−/−* ESCs remain undifferentiated for several passages and *adnpa adnpb* double mutant zebrafish embryos exhibit mild Wnt-related phenotypes.

## Methods

### ES cell culture

Mouse embryonic stem cells (mESCs) R1 were maintained in Dulbecco’s Modified Eagle Medium (DMEM, BI, 01-052-1ACS) high glucose media containing 10% FBS (Gibco, 10099141), 10% knockout serum replacement (KSR, Gibco, 10828028), 1 mM sodium pyruvate (Sigma, S8636), 2 mM L-glutamine (Sigma, G7513), 1000 U/ml leukemia inhibitory factor (LIF, Millipore, ESG1107), and penicillin/streptomycin (Gibco, 15140–122) at 37 °C with 5% CO2.

The commercial ESGRO-2i Medium (Merck-Millipore, SF-016-200) was also used when necessary^36^. We found that in 2i medium, *Adnp−/−* ESCs adopted morphology indistinguishable to that of control ESCs, and maintain self-renewal capacity for more than 20 passages that we tested.

### Embryoid body formation

ESCs differentiation into EBs was performed in attachment or suspension culture in medium lacking LIF or KSR^36^.

### ESC differentiation toward a neural fate

To induce ESC directional neural differentiation, a neurosphere suspension culture system was used. For Stage 1 of neural induction, cells were dissociated and suspended at a density of 1.5 × 10^6^ cells in 10 cm^2^ dish coated with 5 ml 1% agarose. The dissociated cells were cultured with 45% Neurobasal medium (Life Technologies, 21103–049) and 45% DMEM/F12 (BI, 01-172-1ACS) containing 1×B27 (Gibco, 17504044), 1×N2 (Gibco, 17502048), and 20 ng/ml bFGF (Peprotech, AF-100-18B) and 10 ng/ml EGF (Peprotech, 315–09–1000). Cells were cultured on a shaker with low speed at 37 °C in 5% CO_2_ incubator. Half of the culture medium was changed every 2 days. For Stage 2 of neuronal differentiation, the neurospheres were plated onto gelatin-coated six-well plates at day 9, and were cultured for additional 8–10 days in DMEM/F12 containing 2.5% FBS and 1 μM retinoic acid (Sigma, R2625).

MG132 was used to inhibit the proteasome degradation. mESCs or NPCs were treated with 10 μM MG132 (MCE, HY-13259) for 4 h before harvesting cells. For rescue experiments by activating Wnt signaling, mESCs or neurospheres were treated with 25 ng/ml Wnt3a (Peprotech, 315–20–10) or 3 μM CHIR99021 (Selleck, S1263) at the indicated time points during ESC neural induction.

### Lentiviral transduction for shRNA knockdown

The shRNA plasmids for *Adnp* (TRCN0000081670; TRCN0000081671) and the GFP control (RHS4459) were purchased from Dharmacon (USA). To make lentivirus, shRNA plasmids and Trans-lenti shRNA packaging plasmids were co-transfected into H293T cells according to the kit manual (Open Biosystems, TLP4615). After determining the virus titer, mESCs were transduced at a multiplicity of infection of 5:1. Puromycin selection (1 μg/ml) for 4 days was applied to select cells with stable viral integration. qRT-PCR and western blotting were used to assess the knockdown of *Adnp*.

### Generation of *Adnp−/−* ESCs

*Adnp−/−* mESCs were generated by CRISPR/Cas9 technology. Briefly, we designed two gRNAs on exon 4 of *Adnp* gene by using the online website http://crispr.mit.edu/. The gRNAs sequences are: gRNA 1: 5′-CCCTTCTCTTACGAAAAATCAGG-3′; gRNA 2: 5′-CTACTTGGTGCGCTGGAGTTTGG-3′. gRNAs were cloned into the pUC57-U6 expression vector with G418 resistance. The plasmids encoding gRNA and hCas9 were co-transfected into mESCs using Lipofectamine 2000 (Gibco, 11668019). After 48 h, mESCs were selected with 500 μg/ml G418 for 7 days. Then the cells were reseeded on a 10-cm dish coated with 0.1% gelatin to form colonies. The single colony was picked up, trypsinized, and passaged at low density. DNA from single colonies from the passaged cells was extracted and used for genotyping.

### Generation of 3×Flag-tagged *Adnp−/−* mESC cell lines

The full-length *Adnp* cDNA (NM_009628.3) was amplified by PCR and then cloned into the pCMV-3×Flag vector. The full-length *Adnp* cDNA containing N-terminal 3×Flag sequence was subcloned into pCAG-IRES-Puro vector. To make stable transgenic cells, *Adnp−/−* mESCs were transfected with pCAG-IRES-Puro-3×FLAG-*Adnp* vector using Lipofectamine 2000 (Gibco, 11668019). Forty-eight hours later, cells were selected with 1 μg/ml puromycin. After 4–5 days of drug selection, cells were expanded and passaged. Western blot assay was performed to confirm the transgenic cell line using FLAG antibodies.

To make Tet-Express inducible transgenic cells, 3×Flag-*Adnp* or HA-*Ctnnb1* were subcloned into pTRE3G expression vector (Clontech, PT5167-1 and PT3996-5) using the In-Fusion HD Cloning System (Clontech, 638910). Stable transgenic cell lines were established according to the manual of the Tet-Express inducible expression systems (Clontech, 631169). Briefly, *Adnp−/−* ESCs were transfected with 2 μg pTRE3G-3×Flag-*Adnp* or pTRE3G-HA-*Ctnnb1* with linear 100 ng puromycin marker using Lipofectamine 2000 transfection reagent. Ninety-six hours later, 1 μg/ml puromycin was added and drug selection was performed for 2 weeks to establish the stable transgenic cell line. To induce target gene expression, 3 × 10^6^ transgenic cells were plated in six-well plates. The next day, the Tet-Express transactivator (Clontech, 631178) was added (3 μl Tet-Express to a final 100 μl total volume according to the kit manual) for 1 h in serum-free medium to induce the target gene expression. Then cells were allowed to grow in complete medium for an additional 12–24 h before assay for the target protein induction. Western blot were used to assess the target protein expression levels using FLAG or HA antibodies. In the absence of Tet-Express transactivator, pTRE3G provides very low background expression, whereas addition of Tet-Express protein strongly transactivates target genes.

### FACS experiments

Day 6 ESC-derived neurospheres were washed twice with DPBS and were dissociated into single cells with 0.25% trypsin (BI, 03-050-1A). After fixing with 4% paraformaldehyde at room temperature for 10 min, the cells were washed with 0.5% PBSA (0.5% BSA in PBS) and treated with 90% cold methanol for 15 min. After extensive washes, the cells were suspended with 200 μl PBSA and filtered with the flow tube. Then the cells were incubated with anti-NESTIN (Abcam, ab7659), anti-PAX6 (Proteintech, 12323-1-AP) antibodies at room temperature for 15 min. After three-times wash with 0.5% PBSA, the cells were incubated with secondary antibodies (1:500 dilution in PBSA, Alexa Fluor 488) at room temperature for 15 min in the dark. The cells were washed with PBSA for two times and resuspended with 400 μl PBSA, and were sorted by the BD AccuriC6 flow cytometer in the core facility of IHB (performed by Wang Yan). Data were analyzed by FlowJo 10.5.0 (TreeStar, OR, USA).

### RNA preparation, qRT-PCR, and RNA-seq

Total RNA from mESCs, neurospheres, and neuronal cells was extracted with a Total RNA kit (Omega, R6834-01). A total of 1 μg RNA was reverse transcribed into cDNA using the TransScript All-in-One First-Strand cDNA synthesis Supermix (Transgen Biotech, China, AT341). Quantitative real-time PCR was performed using the TransStart Tip Green qPCR SuperMix (Transgen Biotech, China, AQ-141). The primers used for qRT-PCR and cDNA cloning were listed in Tables 1 and 2 in the Supplementary Information. All qRT-PCR experiments were repeated at least three times. The relative gene expression levels were calculated based on the 2^−∆∆Ct^ method. Data were shown as means ± SEM. *p* values were calculated by the two-tailed unpaired *t*-test. The source data for qPCR could be found in the Source Data file.

For RNA-seq, ESCs and day 3, day 4 and day 6 control and mutant ESC-derived neurospheres were collected and treated with Trizol (Invitrogen). RNAs were quantified by a Nanodrop instrument, and sent to BGI Shenzhen (Wuhan, China) for making RNA-seq libraries and deep sequencing. At each time point, at least two biological repeats were sequenced. DEGs were defined by FDR < 0.05 and a Log2 fold change > 1 fold.

### Protein extraction and western blot analysis

For protein extraction, ESCs and neurospheres were harvested and lysed in TEN buffer (50 mM Tris-HCl, 150 mM NaCl, 5 mM EDTA, 1% Triton X-100, 0.5% Na-Deoxycholate, Roche cOmplete Protease Inhibitor). The lysates were quantified by the Bradford method and equal amount of proteins were loaded for western blot assay. Antibodies used for WB were anti-ADNP (R&D Systems, AF5919, 1:1000), anti-non-phospho (Active) β-catenin (Cell Signaling Technology CST, #8814, 1:1000), anti-phospho-β-catenin (Ser33/37/Thr41) (CST, #9561, 1:1000), total β-catenin (CST, #9562, 1:1000), anti-NESTIN (Abcam, ab7659, 1:1000), anti-PAX6 (Proteintech, 12323-1-AP, 1:1000), anti-PCNA (Cusabio, CSB-PA01567A0Rb, 1:1000), anti-GSK3β (Proteintech, 2204-1-AP, 1:1000), anti-pGSK3β (CST, 9336S, 1:1000), anti-PAX2 (Abcam, ab79389, 1:500), anti-TuJ1 (Abcam, ab78078, 1:1000), anti-GFAP (Abcam, ab53554, 1:500), anti-HuC/D (Life Technologies, A21271, 1:1000), anti-β-catenin (Sigma, C7207, 1:1000), Anti-FLAG (Sigma, F3165, 1:1000), anti-MYC antibody (Transgen Biotech, HT101, 1:1000), and anti-HA (Abbkine, A02040, 1:1000). Briefly, the proteins were separated by 10% SDS-PAGE and transferred to a PVDF membrane. After blocking with 5% (w/v) nonfat milk for 1 h at room temperature, the membrane was incubated overnight at 4 °C with the primary antibodies. Then the membranes were incubated with a HRP-conjugated goat anti-rabbit IgG (GtxRb-003-DHRPX, ImmunoReagents, 1:5000), a HRP-linked anti-mouse IgG (7076S, Cell Signaling Technology, 1:5000) for 1 h at room temperature. The GE ImageQuant LAS4000 mini luminescent image analyzer was used for photographing. Western blot experiments were repeated at least two times. The source data for all the western blots could be found in the Source Data file.

Quantification of western blot bands was performed by ImageJ software, according to the website: https://imagej.nih.gov/ij/docs/guide/146-30.html. Briefly, the rectangle tool was selected and used to draw a box around the lane, making sure to include some of the empty gel between lanes and white space outside of the band. All lanes were selected one by one. Once all lanes are defined, go to Plot lanes to generate histograms of each lane. Then the relative values were calculated by dividing each value by the control lane. The value of the control bands was set at 1.

### Co-immunoprecipitation assay (Co-IP)

Co-IPs were performed with the Dynabeads Protein G (Life Technologies, 10004D) according to the manufacturer’s instructions. Briefly, 1.5 mg Dynabeads was conjugated with antibodies or IgG overnight at 4 °C. Antibodies were used are: 10 μg IgG (Proteintech, B900610), or 10 μg anti-ADNP antibody (R&D Systems, A5919), or 10 μg anti-FLAG antibody (Proteintech, 20543-1-AP), or 10 μg anti-HA antibody (Abbkine, A02040), or 10 μg anti-MYC antibody (Transgen Biotech, HT101). The next day, total cell lysates and the antibody-conjugated Dynabeads were incubated overnight at 4 °C with shaking. After three-times washing with PBS containing 0.1% Tween, the beads were boiled at 95 °C for 5 min with the 6×Protein loading buffer and the supernatant was collected for future WB analysis.

### Luciferase reporter assays

The TopFlash-luc reporter was kindly provided by Prof. Zongbin Cui from Institute of Hydrobiology, Wuhan. HEK293T cells (about 1 × 10^5^ cells) were seeded in 24-well plates in DMEM medium containing 10% FBS (Transgen Biotech, FS101-02). After 24 h, the cells were transiently transfected with the indicated luciferase reporters using Liposomal Transfection Reagent (Yeasen, 40802ES03). For mESCs transfection, the Neon Transfection System was used according to the manual. Briefly, the trypsinized mESCs were resuspended in the electroporation buffer at a density of 1 × 10^7^ cells/ml. The TopFlash-luc reporter and pTK-Renilla plasmids were added into the cells. Then electroporation was performed using the Neon Transfection System according to the manufacturer’s instructions. After transfection, the cells were treated with or without 10 ng/ml Wnt3a for 16 h. The luciferase activity was measured with Dual-luciferase Reporter Assay System (Promega, Madison, E1910). Renilla was used as an internal control.

For luciferase reporter assays in zebrafish embryos, one-cell stage wild type and *adnpa adnpb* double mutant embryos were injected with 200 pg TopFlash and 20 pg pTK-Renilla plasmids, and the 24 hpf embryos were collected. Three pools of ten embryos each were lysed with the passive lysis buffer and assayed for luciferase activity according to the Dual Luciferase System (Promega). The TopFlash luciferase activities were measured with the Dual-luciferase Reporter Assay System. All luciferase reporter assays represent the mean ± SEM from three independent measurements of pools. At least three independent experiments were performed in different batches of embryos.

### Immunofluorescence assay

ESC-derived neurospheres were collected and fixed with 4% paraformaldehyde for half an hour at room temperature. Then the cells were washed with PBST (phosphate-buffered saline, 0.1% Triton X-100) three times, each for 15 min. Following the incubation with blocking buffer (5% normal horse serum, 0.1% Triton X-100, in PBS) for 2 h at room temperature, the cells were incubated with primary antibodies at 4 °C overnight. After three-times wash with PBST, the cells were incubated with secondary antibodies (1:500 dilution in blocking buffer, Alexa Fluor 488, Life Technologies) at room temperature for 1 h in the dark. The nuclei were counter-stained with DAPI (Sigma, D9542, 1:1000). After washing with PBS twice, the slides were mounted with 100% glycerol on histological slides. Images were taken by a Leica SP8 laser scanning confocal microscope (Wetzlar, Germany). About ten images were taken for each slide at different magnifications.

Quantification of IF staining was done by ImageJ software^37^. The fluorescence intensity was measured, based on the IF images from at least three biologically independent experiments. When measuring the fluorescence intensity, the entire image or selected area was selected for both the control and mutant group. A total of 3–6 images were measured for each group, and a mean fluorescence intensity value (after subtracting background) was calculated. The control group was arbitrarily set as 1 (or 100%), and the relative fluorescence intensity was expressed as the percentage relative to that of the control group. The results were shown as mean ± SEM. *p* values were calculated by the two-tailed unpaired *t*-test.

### Immunoprecipitation in combination with mass spectrometry

For mass spectrometry analysis, the IP samples (immunoprecipitated by IgG or ADNP antibody) were run on SDS-PAGE gels and stained with the Coomassie Blue. Then the entire lanes for each IP samples were cut off and transferred into 15 ml tubes containing deionized water. The treatment of the samples and the mass spectrometry analysis were done by GeneCreate Biological Engineering Company (Wuhan, China).

### Protein–protein interaction assay

Protein–protein interaction assay was performed using a rabbit reticulocyte lysate system (Promega, L5020, USA)^38^. Tagged-ADNP, tagged-ADNP mutants, tagged-β-catenin, and tagged-β-catenin mutants were synthesized using the TNT coupled reticulocyte lysate system according to the manual. Briefly, 1 μg of circular PCS2-version of plasmids was added directly to the TNT lysates and incubated for 1.5 h at 30 °C. 1 μl of the reaction products was subjected to WB assay to evaluate the synthesized protein. For protein–protein interaction assay, 5–10 μl of the synthesized HA- or FLAG-tagged proteins was mixed in a 1.5 ml tube loaded with 300 μl TEN buffer, and the mixture was shaken for 30 min at room temperature. Next, IP or pull-down assay was performed using Dynabeads protein G coupled with anti-FLAG or anti-HA antibodies as described above.

### Zebrafish maintenance

Zebrafish (*Danio rerio*) was maintained at 28.5 °C on a 12 h light/12 h dark cycle. All procedures were performed with the approval of the Institute of Hydrobiology, Chinese Academy of Sciences, Wuhan, China.

### Generation zebrafish *adnp* mutants by CRISPR/Cas9

Zebrafish *adnp* mutants were generated by CRISPR/Cas9 technology. The gRNA targeting sequences for *adnpa* and *adnpb* gene were 5′-GGACTCTGGAAACCCACGTC-3′ and 5′-GGAGGACTTCATGGGGCAG-3′, respectively. gRNAs were generated using the MEGAshortscript T7 kit (Thermo Fisher). The Cas9 mRNA was synthesized using the mMESSAGE mMACHINE SP6 Kit (AM1340, Thermo Fisher). The mixture containing 200 ng/µl Cas9 mRNA and 80 ng/µl gRNA was co-injected into one-cell stage zebrafish embryos. The genomic DNA of 20 injected embryos at 24 hpf was extracted and subjected to PCR amplification. The DNA fragments containing the gRNA targeting sequences were amplified by PCR using primers 5′-TGCTCAGATTGCCCGTTT-3′ and 5′-GATAGGTGCACTTCTTGCAGTA-3′ for *adnpa* gene, 5′-AATGTGCACAGCGAGGACTT-3′ and 5′-GACAAGGACTGTGTAGCCCC-3′ for *adnpb* gene, respectively. The genotype was confirmed by DNA sequencing. Adults raised up from injected embryos were screened for mosaic founders by the amplicon sequencing. The mosaic founders were outcrossed to wild type to obtain the F1 offsprings with stable germline transmission. The F1 heterozygous carrying 7 bp deletion (*adnpa*) and 5 bp deletion (*adnpb*) were outcrossed to wild type to generate F2 heterozygous, respectively. The F2 heterozygous were inter-crossed to generate homozygous *adnpa*−/− and *adnpb*−/−, respectively. The *adnpa−/−* and *adnpb−/−* adults were outcrossed to each other to generate *adnpa* and *adnpb* double mutant. The subsequent screening was performed by high resolution melt analysis using primers 5′-TTCCGCAATGTTCACAGGGA-3′ and 5′-GGCATATGGAAGAGCCTGACG-3′ for *adnpa*, 5′-CACATCAGGTTGTTTCATATGCCT-3′ and 5′-TCACGTGTCTTTTCCAGACGA-3′ for *adnpb*, respectively.

### Microinjections into zebrafish embryos

For overexpression experiments, full-length cDNA encoding Adnpa (NC_007122.7) was amplified and cloned into the PCS2 (+) vector. The primers we used are GATGTTTCAGCTTCCAGTGAATAACC (forward) and CACAAGCCATCATCTACCAAGC (reverse). The *Adnpa* mRNAs were transcribed with the mMESSAGE mMACHINE SP6 Kit (Invitrogen, AM1340) and were dissolved in nuclease-free water to the final concentration of 150 ng/μl. A total of 1–5 nl mRNAs were microinjected into one-cell stage wild-type zebrafish embryo using the WPI microinjector (WPI, USA). 1–2 dpf embryos were anesthetized in 0.016% Tricaine and photographed under a Leica stereomicroscope.

### Whole-mount immunofluorescence in zebrafish

Briefly, 12–48 hpf zebrafish embryos were fixed in 4% paraformaldehyde overnight at 4 °C and then underwent proteinase K treatment for 8 min at RT. The primary antibodies were anti-HuC/HuD monoclonal antibody (Life Technologies, USA, 1:350 dilution in PBST), anti-FLAG (F3165, Sigma), and anti-β-catenin (20543-1-AP, Proteintech). After blocking for 2 h at room temperature with solution (0.1% Triton X-100, 1% BSA, and 1% DMSO in PBS), the embryos were incubated overnight with the primary antibodies at 4 °C with shaking. After three-times wash with PBST, embryos were incubated with the secondary antibodies (1:1000, Goat anti-Mouse IgG secondary antibody, Alexa Fluor 488, Life technologies, USA) for 2 h at RT. Finally, the embryos were counter-stained with DAPI. Embryos were imaged in 4% methyl cellulose using a Leica SP8 Confocal microscope.

### Whole-mount in situ hybridization (WISH)

WISH was performed for RNA expression analysis in zebrafish embryos^38^. For making the RNA probes, total RNA was extracted from zebrafish embryos, and cDNAs were made by RT-PCR. Templates used for making RNA probes were amplified by PCR using primers containing SP6 or T7 promoters. Primers we used are listed in Table 3 in the Supplementary Information. The DIG-labeled anti-sense probes for *adnpa*, *adnpb*, *dlx5a*, *neurod1*, and *phox2a* were generated using the DIG RNA Labeling Kit (SP6/T7) (Roche). Zebrafish embryos at different developmental stages were collected and fixed with 4% PFA overnight at 4 °C. Following the WISH, the embryos were transferred to six-well plates and submerged in 100% glycerol for imaging.

### Quantification, statistics, and reproducibility

All experiments were repeated at least two times. Data are presented as mean values ± SEM unless otherwise stated. *p* values were calculated by the two-tailed unpaired *t*-test. Differences in means were statistically significant when *p* < 0.05, with exact *p* value being provided.

### Reporting summary

Further information on research design is available in the Nature Research Reporting Summary linked to this article.

## Supplementary information

supplementary information

peer review

Reporting Summary

## Data Availability

Raw data for RNA-seq and IP-followed mass spectrometry are available on public database at https://bigd.big.ac.cn/gsa/. The accession numbers are CRA002148 (day 0), CRA001624 (ESC state), and CRA001901 (day 3 and day 6 of ESC neural differentiation). The source data underlying Figs. 1c, e, h; 2c, g, j; 4c–e; 5b–j; 6a–i; 7b–f; 9c, f, g and Supplementary Figs. 1d, f; 2a; 4c; 5a–c; 6a–e are provided as a Source Data file. All other data will be available upon request. Source Data are provided with this paper.

## Source data

source data
