# Supplementary material for: ADNP promotes neural differentiation by modulating Wnt/β-catenin signaling
Source: Nat Commun. 2020 Jun 12;11:2984. doi: 10.1038/s41467-020-16799-0 (PMC7293280; doi:10.1038/s41467-020-16799-0)
Supplement: Supplementary file 1 — supplementary information [file 41467_2020_16799_MOESM1_ESM.pdf]

Supplementary information

ADNP promotes neural differentiation by modulating Wnt/β-catenin signaling

Sun et al.,

The supplementary information contains:
1) Supplementary figures

2) Legends for supplementary figures

3) Tables 1-3

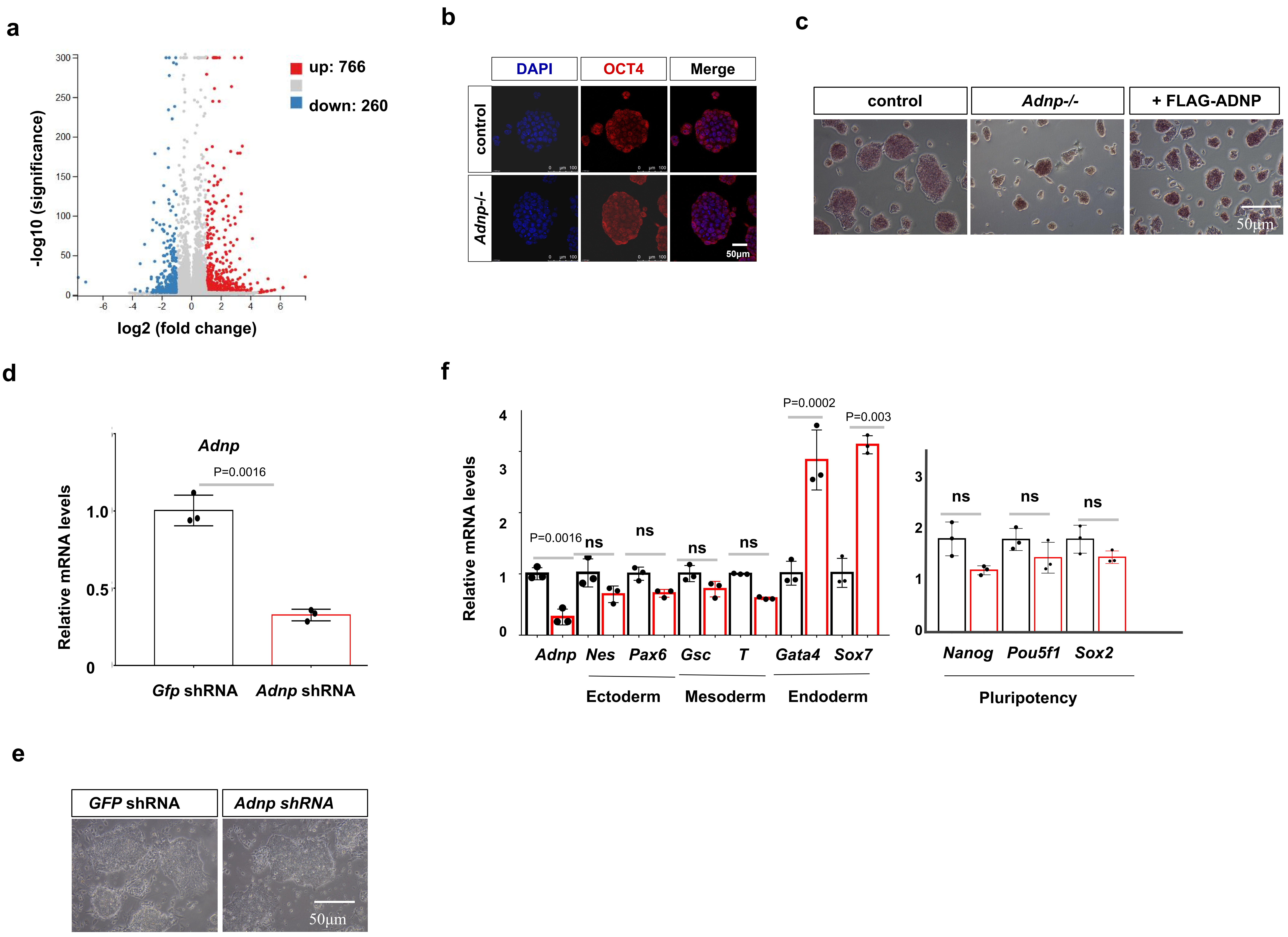
a
-log10 (significance)
log2 (fold change)
up: 766
down: 260
b
DAPI
OCT4
Merge
control
Adnp-/-
50μm
c
control
Adnp-/-
+ FLAG-ADNP
50μm
d
Adnp
P=0.0016
Relative mRNA levels
Gfp shRNA
Adnp shRNA
e
GFP shRNA
Adnp shRNA
50μm
f
Relative mRNA levels
P=0.0016
ns
P=0.0002
P=0.003
Adnp
Nes
Pax6
Gsc
T
Gata4
Sox7
Ectoderm
Mesoderm
Endoderm
Nanog
Pou5f1
Sox2
Pluripotency

Supplementary Fig. 1. Generation and characterization of *Adnp-/-* ESCs. **a** Valcano plot showing the up- and down-regulated genes in the RNA-seq analysis of control and *Adnp-/-* ESCs. The RNA-seq experiments were repeated two times. DEGs were defined by FDR < 0.05 and a Log2 fold change > 1. **b** IF staining of OCT4 for control and *Adnp-/-* ESCs. **c** Alkaline phosphotase staining for long-term passaged control, *Adnp-/-* ESCs and FLAG-ADNP restoring *Adnp-/-* ESCs (*Adnp-/-* ESCs showed abnormal morphology and alkaline phosphotase activities after passaged for 6-10 times in LIF/KSR medium). **d** Relative *Adnp* mRNA levels for *Gfp* shRNA and *Adnp* shRNA knockown ESCs (n=3 per group). **e** Representative image showing morphology of control and early passaged *Adnp* shRNA ESCs. **f** The expression of selected pluripotency-related, mesodermal, neuroectodermal and endodermal genes in control and early passaged *Adnp* shRNA ESCs. qRT-PCR was based on 3 biologically independent experiments (n=3 per group). Data are presented as mean values +/- SEM in d and f. p values by two-tailed unpaired t test are shown in d and f. ns: not significant. Similar results were obtained of at least two biologically independent experiments and shown are the representative images for b, c and e.

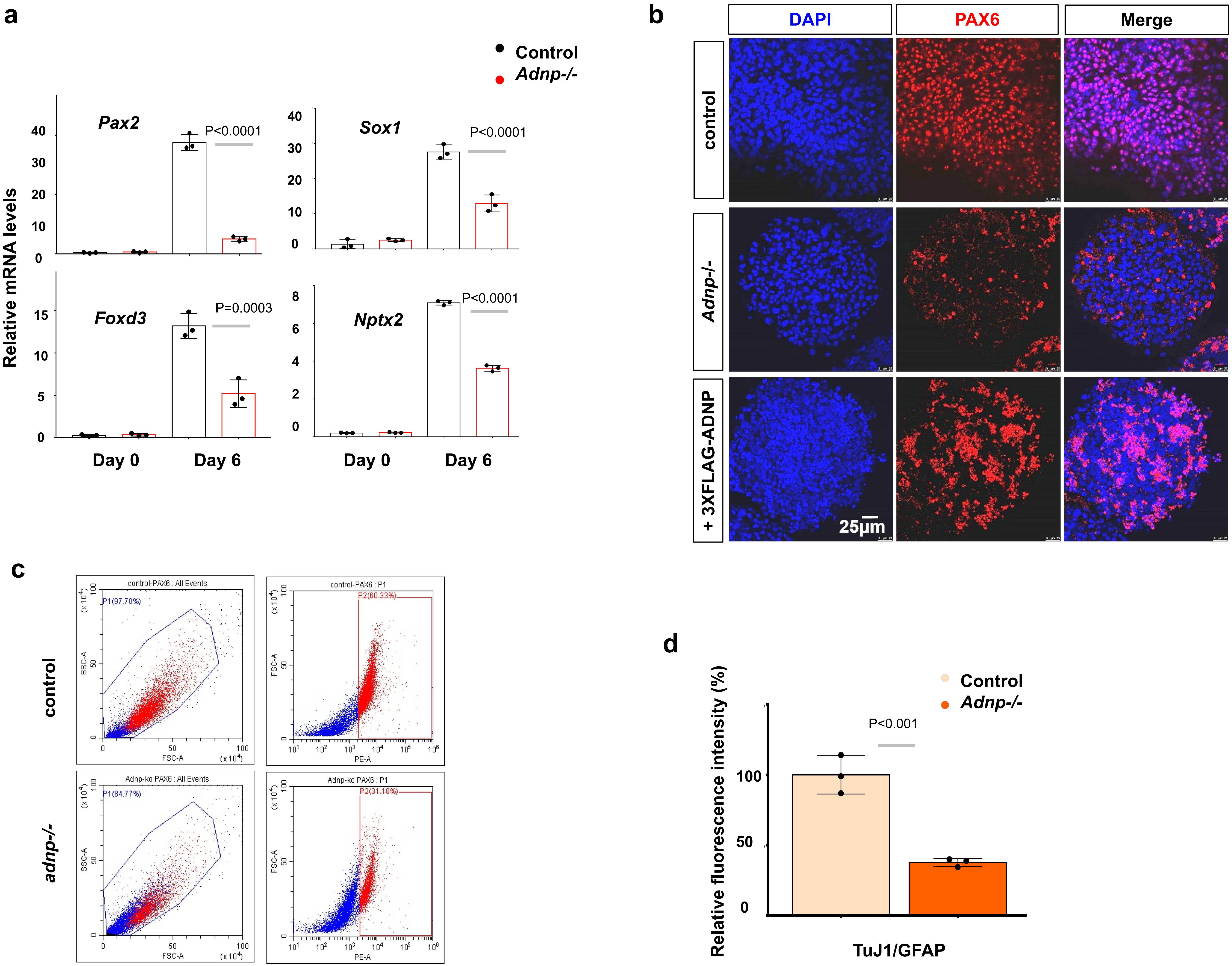

a
Control
Adnp-/-
Relative mRNA levels
Pax2
P<0.0001
Sox1
P<0.0001
Foxd3
P=0.0003
Nptx2
P<0.0001
Day 0
Day 6
b
DAPI
PAX6
Merge
control
Adnp-/-
+ 3XFLAG-ADNP
25μm
c
control
adnp-/-
control-PAX6 : All Events
P1(97.70%)
control-PAX6 : P1
P2(60.33%)
Adnp-ko PAX6 : All Events
P1(84.77%)
Adnp-ko PAX6 : P1
P2(31.18%)
SSC-A
FSC-A
PE-A
d
Control
Adnp-/-
P<0.001
Relative fluorescence intensity (%)
TuJ1/GFAP

Supplementary Fig. 2 . ADNP is required for proper ESC neural differentiation. a The expression of representative neural genes at indicated time points. qRT-PCR was based on 3 biologically independent experiments (n=3 per group). Data are presented as mean values +/- SEM, and *p* values by two-tailed unpaired t test are shown. **b** IF staining of PAX6 for day 6 control, *Adnp-/-* ESC and FLAG-ADNP restoring *Adnp-/-* ESC-derived neurospheres. 3 biologically indepedent experiments were performed. Similar results were obtained and shown are represenrtative images. **c** Gating strategy for detection of PAX6 positive cells of panel d in Figure 2. **d** Quantification of mean fluorescence intensity of TuJ1 and GFAP double staining using ImageJ, for day 19 control and *Adnp-/-* ESC-derived neuronal cell cultures. Mean fluorescence intensity was calculated based on 3 biologically independent experiments (n=3-5 different regions of interest per group). Data are presented as mean values +/- SEM and *p* values by two-tailed unpaired t test are shown.

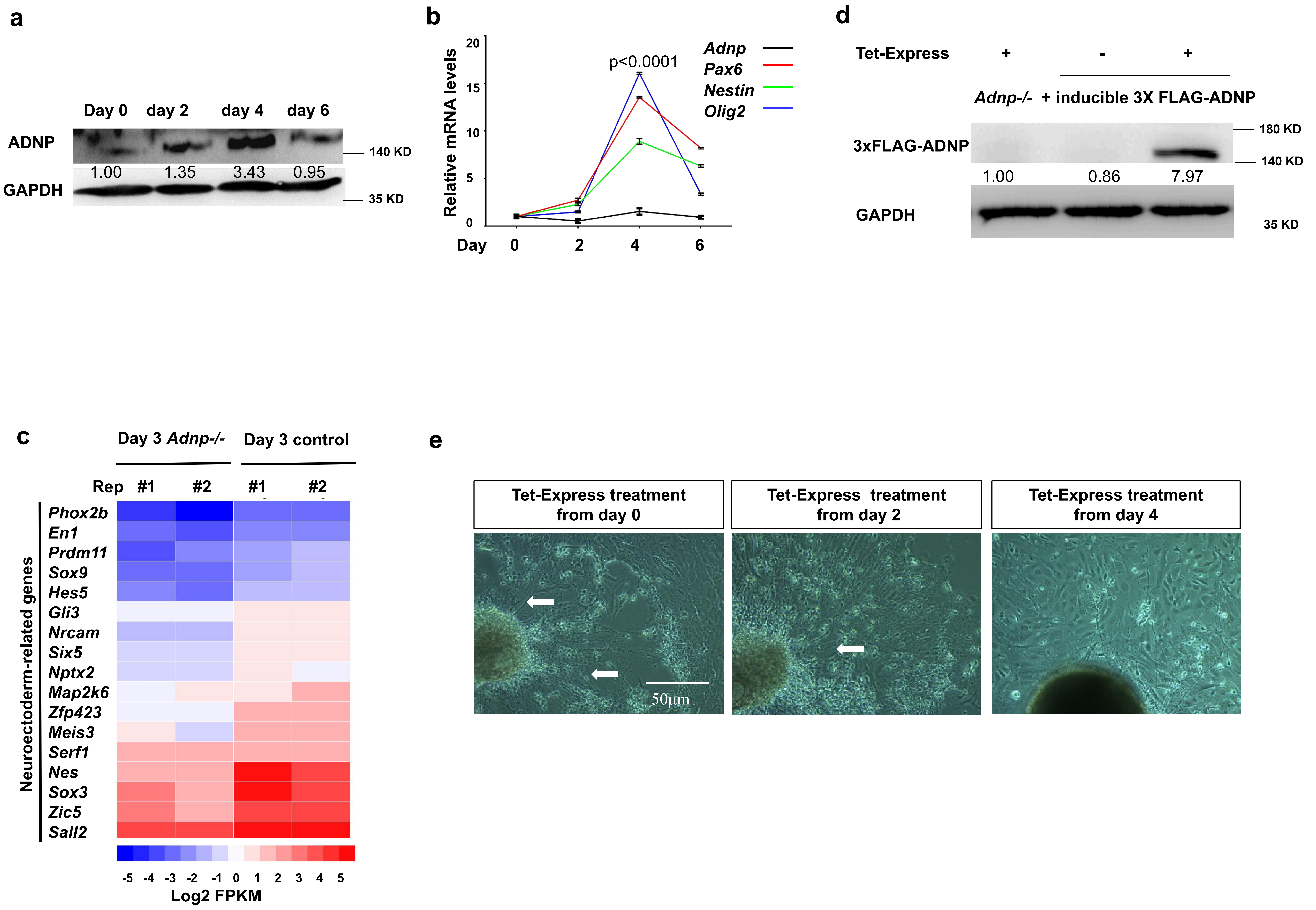
a
Day 0
day 2
day 4
day 6
ADNP
140 KD
1.00
1.35
3.43
0.95
GAPDH
35 KD
b
Relative mRNA levels
20
15
10
5
0
p<0.0001
Adnp
Pax6
Nestin
Olig2
Day
0
2
4
6
d
Tet-Express
+
-
+
Adnp-/-
+ inducible 3X FLAG-ADNP
3xFLAG-ADNP
180 KD
140 KD
1.00
0.86
7.97
GAPDH
35 KD
c
Day 3 Adnp-/-
Day 3 control
Rep
#1
#2
#1
#2
Neuroectoderm-related genes
Phox2b
En1
Prdm11
Sox9
Hes5
Gli3
Nrcam
Six5
Nptx2
Map2k6
Zfp423
Meis3
Serf1
Nes
Sox3
Zic5
Sall2
-5
-4
-3
-2
-1
0
1
2
3
4
5
Log2 FPKM
e
Tet-Express treatment from day 0
Tet-Express treatment from day 2
Tet-Express treatment from day 4
50μm

Supplementary Fig. 3. ADNP promotes the expression of neuroectoderm developmental genes. Related to Figure 3. **a** Dynamic expression profile of ADNP during first 6 days of neural induction of control ESCs. **b** Dynamic expression profile of *Adnp*, *Pax6*, *Nestin* and *Olig2* genes during the first 6 days of neural induction of wild type ESCs. qRT-PCR was based on 3 biologically independent experiments (n=3 per group). Data are presented as mean values +/- SEM (two-tailed unpaired t test: p values are shown). **c** Heat map illustrating the expression of selected neuroectoderm genes that were shown as log2 FPKM in day 3 control and *Adnp-/-* ESC-derived neurospheres. Each lane corresponds to an independent biological RNA-seq sample. **d** WB showing that addition of Tet-Express transactivated 3×FLAG-ADNP in *Adnp-/-* ESCs. **e** Representative morphology showing neuronal fibre structure in *Adnp-/-* ESC-derived day 19 neuronal cell types that treated with Tet-Express from day 0, day 2 and day 4 of *Adnp-/-* ESC neural induction. The white arrows showing the neuronal fibre structures. The experiments were repeated at least two times, and shown are the representative image.

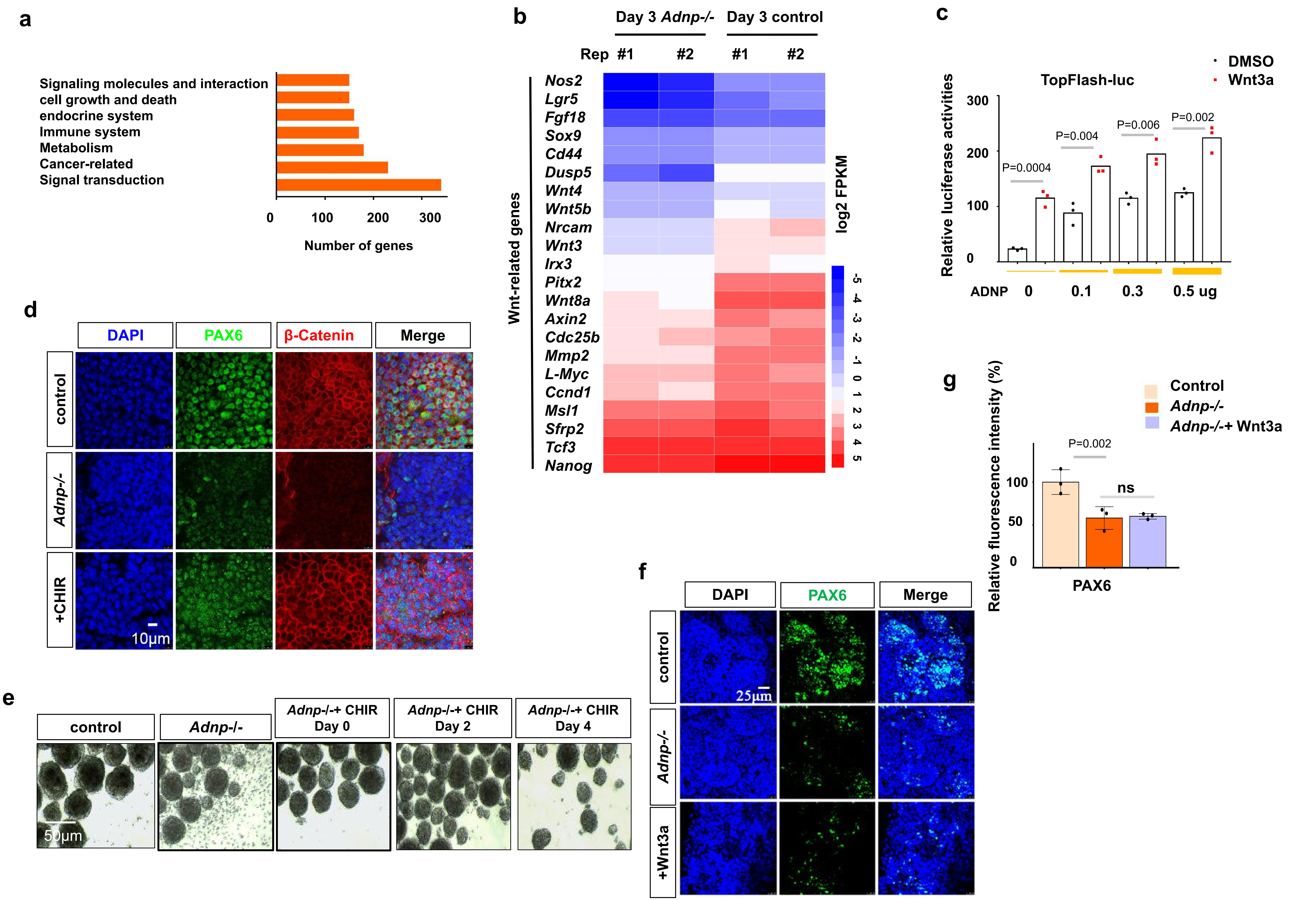

a
Signaling molecules and interaction
cell growth and death
endocrine system
Immune system
Metabolism
Cancer-related
Signal transduction
0 100 200 300
Number of genes
b
Day 3 Adnp-/-
Day 3 control
Rep #1 #2 #1 #2
Wnt-related genes
Nos2
Lgr5
Fgf18
Sox9
Cd44
Dusp5
Wnt4
Wnt5b
Nrcam
Wnt3
Irx3
Pitx2
Wnt8a
Axin2
Cdc25b
Mmp2
L-Myc
Ccnd1
Msl1
Sfrp2
Tcf3
Nanog
log2 FPKM
-5 -4 -3 -2 -1 0 1 2 3 4 5
c
TopFlash-luc
DMSO
Wnt3a
Relative luciferase activities
300
200
100
0
P=0.0004
P=0.004
P=0.006
P=0.002
ADNP 0 0.1 0.3 0.5 ug
d
DAPI
PAX6
β-Catenin
Merge
control
Adnp-/-
+CHIR
10μm
e
control
Adnp-/-
Adnp-/-+ CHIR Day 0
Adnp-/-+ CHIR Day 2
Adnp-/-+ CHIR Day 4
50μm
f
DAPI
PAX6
Merge
control
Adnp-/-
+Wnt3a
25μm
g
Control
Adnp-/-
Adnp-/-+ Wnt3a
Relative fluorescence intensity (%)
P=0.002
ns
100
50
0
PAX6

Supplementary Fig. 4. Wnt signaling is impaired in the absence of ADNP. **a** KEGG analysis of of DEGs from day 3 and day 6 control and *Adnp-/-* ESC-derived neurospheres, showing enrichment of top 7 pathways related to signaling transduction. **b** Heat map illustrating the expression of selected Wnt-related genes that were shown as log2 FPKM in day 3 control and *Adnp-/-* ESC-derived neurospheres. Each lane corresponds to an independent biological RNA-seq sample. **c** TopFlash luciferase activity analysis of 293T cells transfected with increasing dose of plasmids encoding ADNP, in the absence or presence of Wnt3a. Data are based on two experimental replicates and presented as mean values +/- SEM, and *p* values by two-tailed unpaired t test are shown. **d** Rescue of PAX6 expression by addition of CHIR. Representative IF staining showing PAX6 signal for day 6 control and *Adnp-/-* ESC-derived neurospheres. **e** Representative morphology of day 6 *Adnp-/-* ESC-derived neurospheres after addition of CHIR from the indicated time points. The experiments were repeated two times and similar results were obtained. **f** Rescue of PAX6 expression by addition of 20 ng/ml Wnt3a. Representative IF staining showing PAX6 signals for day 6 control and *Adnp-/-* ESC-derived neurospheres. **g** Quantification of mean fluorescence intensity of PAX6 staining using ImageJ for panel (**f**), based on 3 biologically independent experiments (n=3-5 different regions of interest per group). Data are presented as mean values +/- SEM and *p* values by two-tailed unpaired t test are shown. ns: not significant.

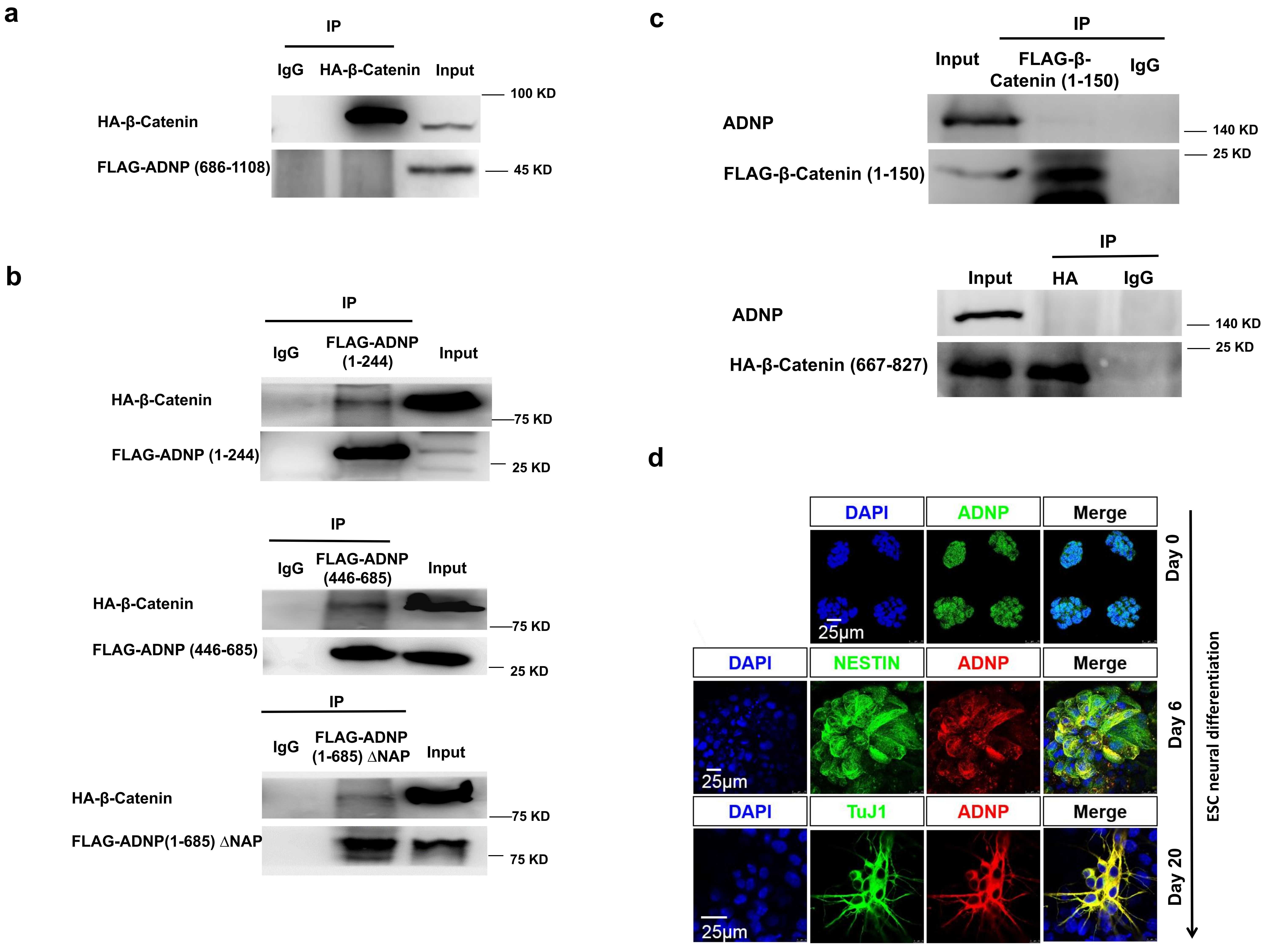
a
IP
IgG
HA-β-Catenin
Input
100 KD
HA-β-Catenin
FLAG-ADNP (686-1108)
45 KD
b
IP
IgG
FLAG-ADNP (1-244)
Input
HA-β-Catenin
75 KD
FLAG-ADNP (1-244)
25 KD
IP
IgG
FLAG-ADNP (446-685)
Input
HA-β-Catenin
75 KD
FLAG-ADNP (446-685)
25 KD
IP
IgG
FLAG-ADNP (1-685) ∆NAP
Input
HA-β-Catenin
75 KD
FLAG-ADNP(1-685) ∆NAP
75 KD
c
IP
Input
FLAG-β-Catenin (1-150)
IgG
ADNP
140 KD
25 KD
FLAG-β-Catenin (1-150)
IP
Input
HA
IgG
ADNP
140 KD
25 KD
HA-β-Catenin (667-827)
d
DAPI
ADNP
Merge
Day 0
25μm
DAPI
NESTIN
ADNP
Merge
Day 6
25μm
DAPI
TuJ1
ADNP
Merge
Day 20
25μm
ESC neural differentiation

Supplementary Fig. 5 Identifying β-Catenin as an ADNP interacting protein. **a** WB showing that the C-terminal of ADNP (686-1108) does not interact with β-Catenin in HEK293T cells. **b** Dissection of FLAG-ADNP-Nter (1-685) that is responsible to interact with β-Catenin in 293T cells. ADNP fragment 1-224, 446-685 both can interact with β-Catenin (the up and middle panels). And ADNP-Nter (1-685) without NAP can still interact with β-Catenin (the bottom panel). **c** WB showing that β-Catenin (1-150) barely interacts with ADNP in HEK293T cells (up), and that β-Catenin (667-827) barely interacts with ADNP in HEK293T cells (down). **d** IF staining of ADNP showing that during ESC neural differentiation ADNP translocates from the nuclei to the cytoplasm, and that ADNP was co-localized with NESTIN and TuJ1 in day 6 ESC-derived neurospheres and day 19 neuronal cell types, respectively. All WB and IF staining experiments were repeated at least two times. Simliar results were obtained and shown are representative images.

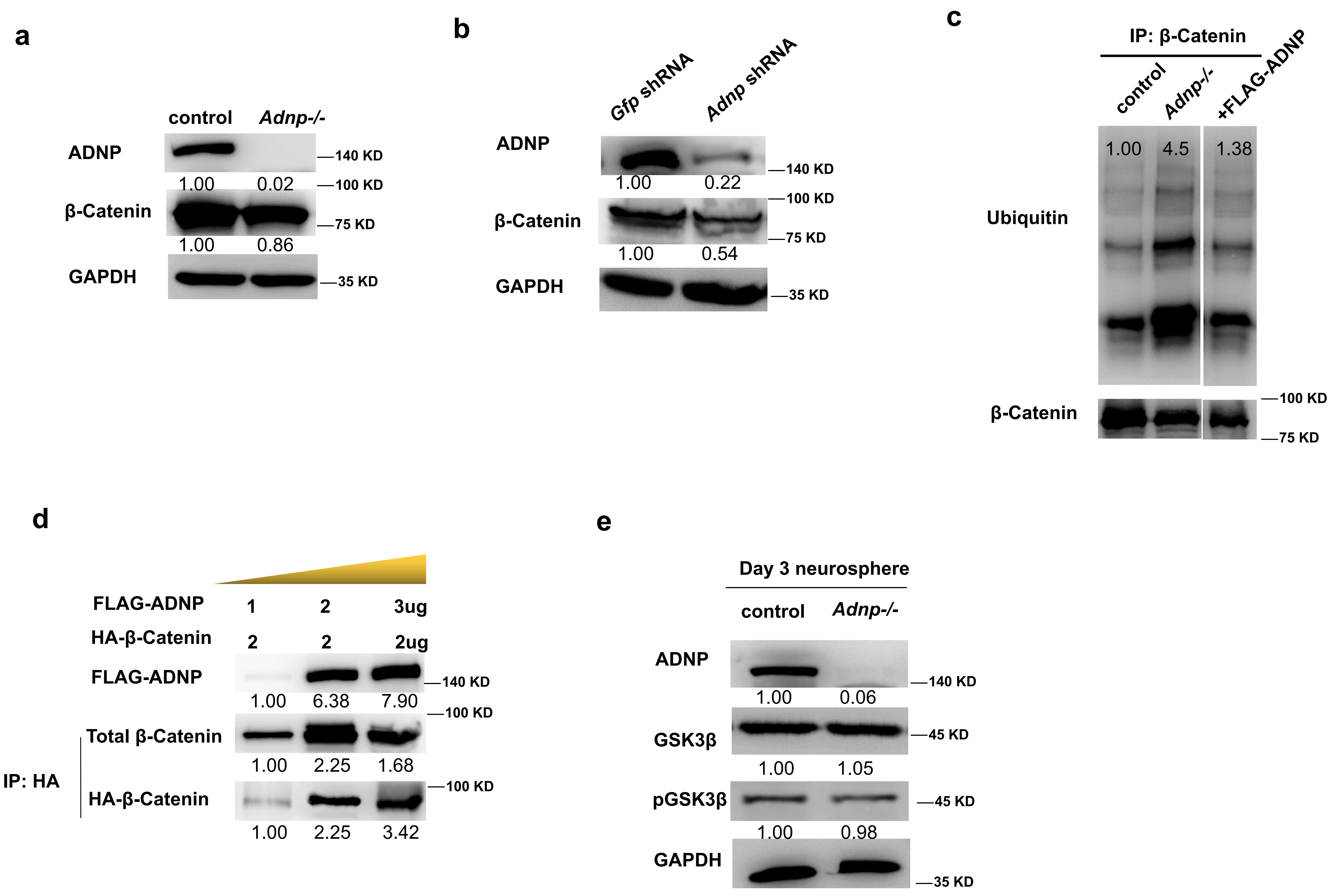
a
control
Adnp-/-
ADNP
140 KD
1.00
0.02
100 KD
β-Catenin
75 KD
1.00
0.86
GAPDH
35 KD
b
Gfp shRNA
Adnp shRNA
ADNP
140 KD
1.00
0.22
100 KD
β-Catenin
75 KD
1.00
0.54
GAPDH
35 KD
c
IP: β-Catenin
control
Adnp-/-
+FLAG-ADNP
1.00
4.5
1.38
Ubiquitin
β-Catenin
100 KD
75 KD
d
FLAG-ADNP
1
2
3ug
HA-β-Catenin
2
2
2ug
FLAG-ADNP
140 KD
1.00
6.38
7.90
100 KD
Total β-Catenin
1.00
2.25
1.68
IP: HA
HA-β-Catenin
100 KD
1.00
2.25
3.42
e
Day 3 neurosphere
control
Adnp-/-
ADNP
140 KD
1.00
0.06
GSK3β
45 KD
1.00
1.05
pGSK3β
45 KD
1.00
0.98
GAPDH
35 KD

Supplementary Fig. 6 ADNP stabilizes β-Catenin during ESC neural differentiation. **a** Representative WB showing the total β-Catenin levels in control and *Adnp-/-* ESCs. **b** WB showing the total β-Catenin levels in day 3 control and *Adnp* shRNA knockdown ESC-derived neurospheres. **c** Representative WB showing the ubiquitylation levels of β-Catenin in day 3 control, *Adnp-/-* and FLAG-ADNP overexpressing *Adnp-/-* ESC-derived neurospheres. **d** Representative WB showing the effects of an increasing dose of ADNP on the co-transfected HA-β-Catenin and total β-Catenin levels. **e** Representative WB showing the effect of ADNP depletion on GSK3β and phosphorylated GSK3β levels. All WB and IP experiments were repeated at least two times. Similar results were obtained, and shown are representative images.

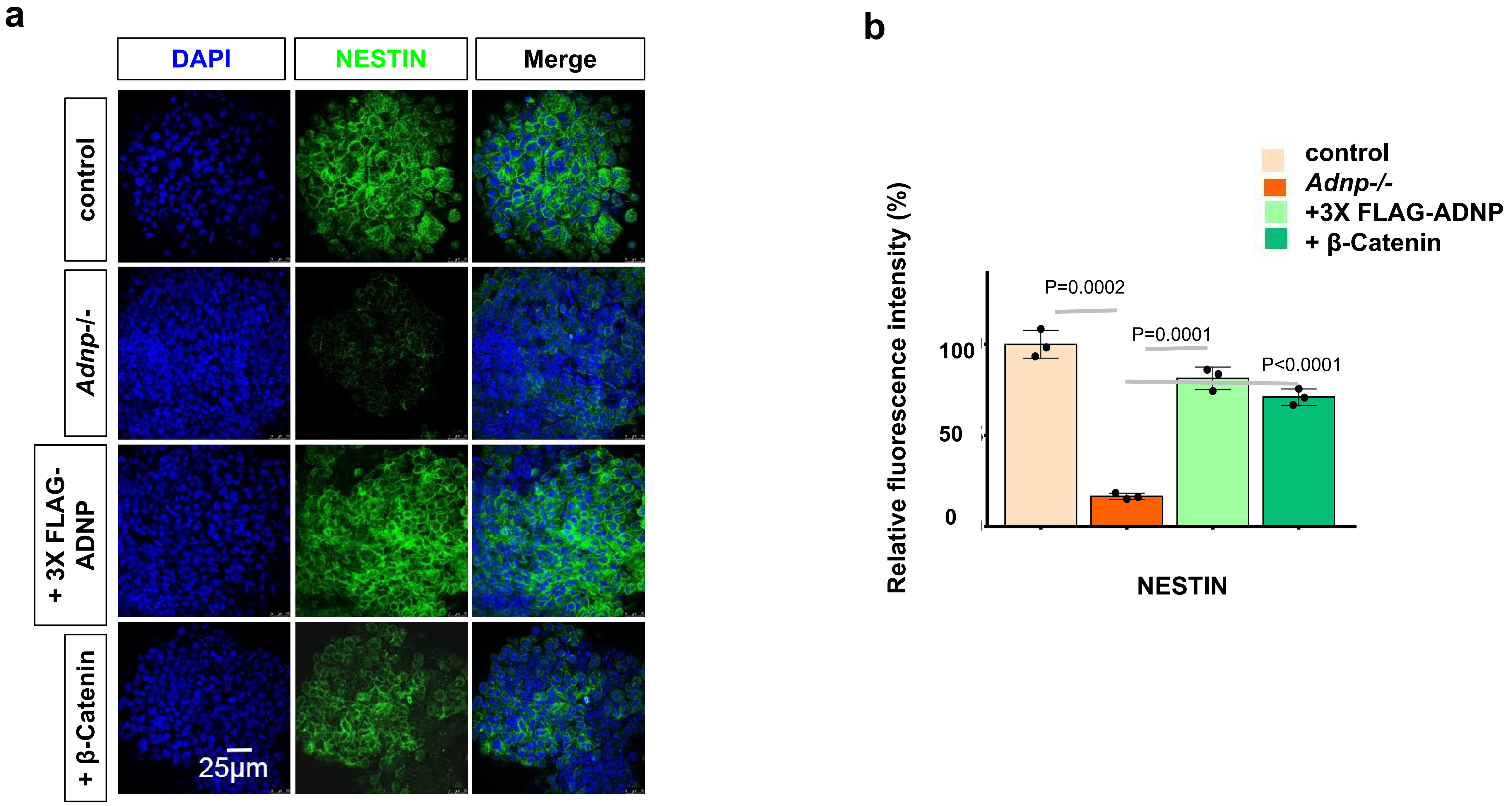
a
DAPI
NESTIN
Merge
control
Adnp-/-
+ 3X FLAG-ADNP
+ β-Catenin
25μm
b
control
Adnp-/-
+3X FLAG-ADNP
+ β-Catenin
P=0.0002
P=0.0001
P<0.0001
100
50
0
Relative fluorescence intensity (%)
NESTIN

Supplementary Fig. 7 Related to Figure 7. **a** IF staining showing the rescue of NESTIN levels in day 6 *Adnp-/-* ESC-derived neurospheres by restoring FLAG-ADNP in ESCs or adding the Tet-Express transactivator at early stage of neural induction. **b** Quantification of mean fluorescence intensity of NESTIN staining using ImageJ for panel (**a**), based on 3 biologically independent experiments (n=3-5 different regions of interest per group). Data are presented as mean values +/- SEM and *p* values by two-tailed unpaired t test are shown. The mean fluorescence intensity of $NESTIN^{+}$ staining in *Adnp-/-* ESC-derived neurospheres was significantly increased after restoring FLAG-ADNP or treatment with the Tet-Express, respectively.

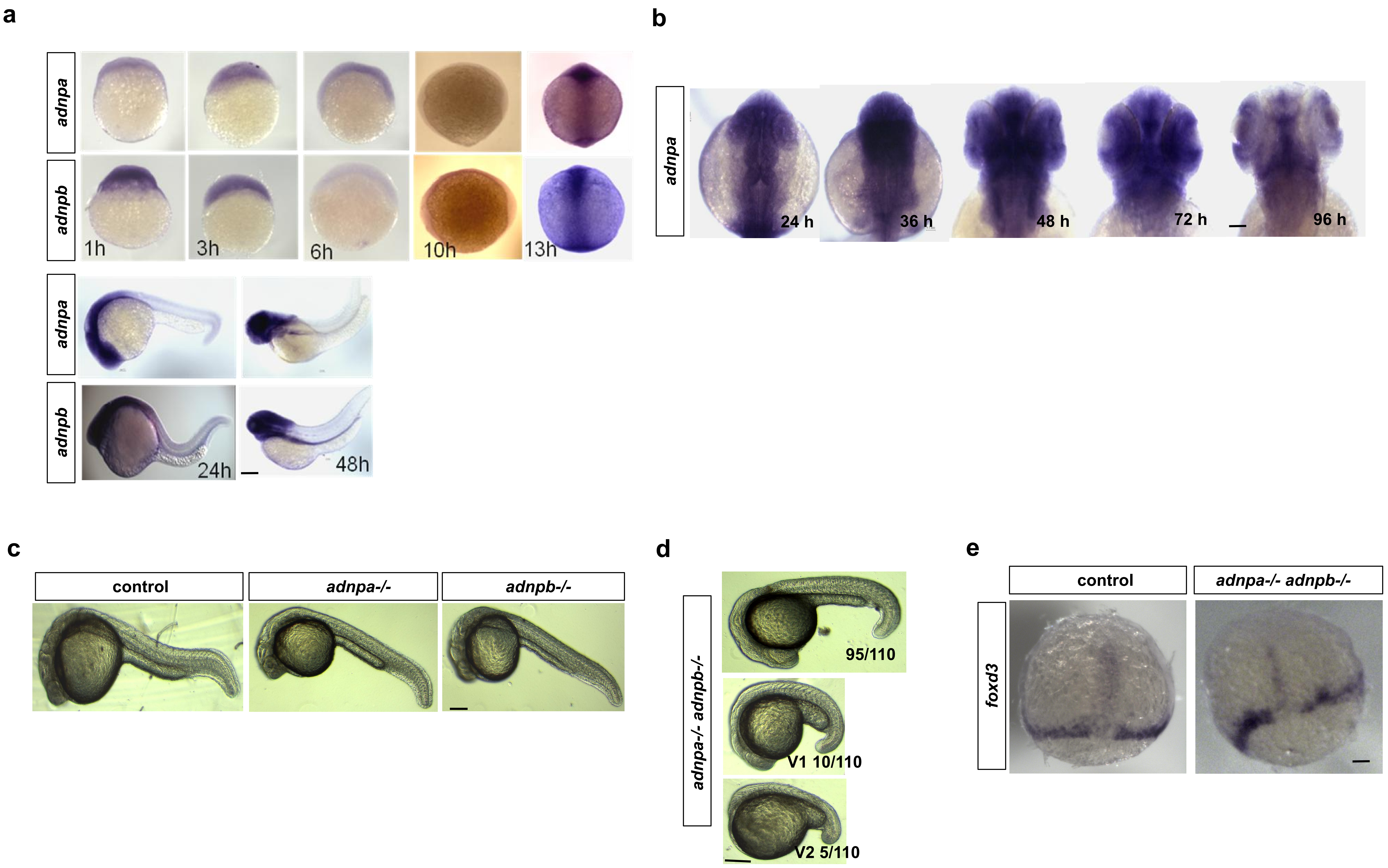
a
adnpa
adnpb
1h
3h
6h
10h
13h
adnpa
adnpb
24h
48h
b
adnpa
24 h
36 h
48 h
72 h
96 h
c
control
adnpa-/-
adnpb-/-
d
adnpa-/- adnpb-/-
95/110
V1 10/110
V2 5/110
e
control
adnpa-/- adnpb-/-
foxd3

Supplementary Fig. 8 Loss of adnp leads to the defective neural development in zebrafish embryos. **a** In situ hybridization results showing the expression of *adnpa* and *adnpb* in embryos at different developmental stages. **b** In situ hybridization results showing *adnpa* expression in head region of embryos at different developmental stages. Dorsal view of head region. **c** Representative morphology of 1 dpf control, *adnpa-/-* and *adnpb-/-* zebrafish embryos. **d** Representative morphology of 1 dpf *adnpa-/- adnpb-/-* embryos. The ventralized phenotypes (V1-V2) were according to the DV patterning index[36]. **e** WISH images for *foxd3* for 8 hpf control and double mutant embryos. WISH experiments were repeated at least two times. Scale bars: 200 µm in a-d, 50 µm in e.

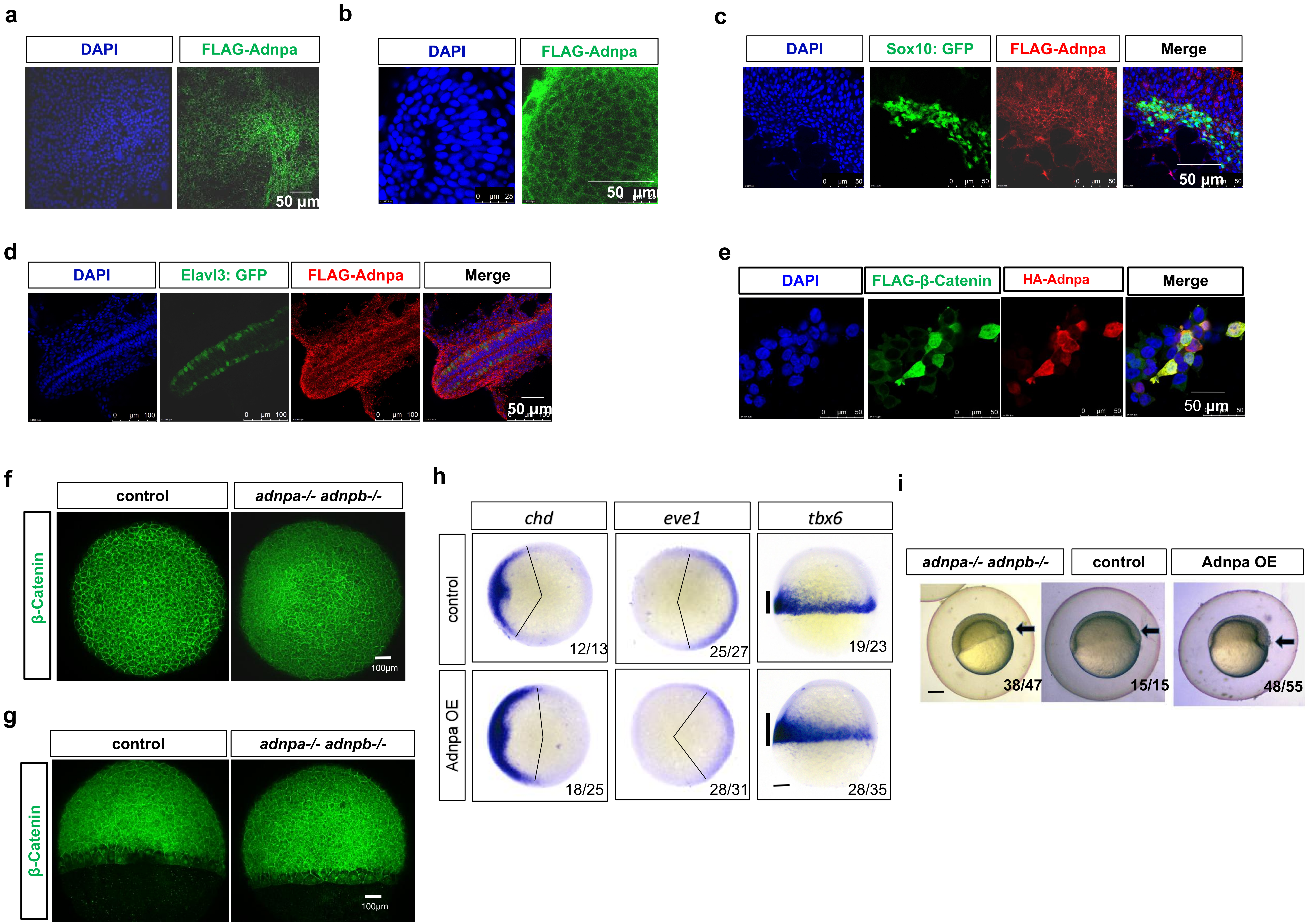
a
DAPI
FLAG-Adnpa
50 μm
b
DAPI
FLAG-Adnpa
50 μm
c
DAPI
Sox10: GFP
FLAG-Adnpa
Merge
50 μm
d
DAPI
Elavl3: GFP
FLAG-Adnpa
Merge
50 μm
e
DAPI
FLAG-β-Catenin
HA-Adnpa
Merge
50 μm
f
control
adnpa-/- adnpb-/-
β-Catenin
100μm
g
control
adnpa-/- adnpb-/-
β-Catenin
100μm
h
chd
eve1
tbx6
control
Adnpa OE
12/13
25/27
19/23
18/25
28/31
28/35
i
adnpa-/- adnpb-/-
control
Adnpa OE
38/47
15/15
48/55

Supplementary Fig. 9 Loss of adnp leads to reduced β-Catenin levels and Wnt signaling in zebrafish embryos. **a** Confocal image showing the cytoplasmic localization of FLAG-Adnpa in 13 hpf embryos. **b** Confocal image showing the cytoplasmic localization of FLAG-Adnpa in 24 hpf embryos. **c** Confocal image showing the co-localization of FLAG-Adnpa and Sox10: GFP in 13 hpf *Tg* (*sox10:gfp*) embryos. Note that FLAG-Adnpa is predominantly localized in the cytoplasm. **d** Confocal image showing the co-localization of FLAG-Adnpa and HuC: GFP in 24 hpf *Tg* (*elavl3:gfp*) embryos. Note that FLAG-Adnpa is predominantly localized in the cytoplasm. **e** IF staining of FLAG and HA showing co-localization of HA-Adnpa and FLAG-β-Catenin in 293T cells. **f** IF staining of β-Catenin showing β-Catenin levels and its cellular localization in 3.5 hpf control and *adnpa-/- adnpb-/-* embryos. Animal view. **g** IF staining of β-Catenin showing β-Catenin levels and its cellular localization in 6 hpf control and *adnpa-/- adnpb-/-* embryos. Straight facing to the dorsal embryos. **h** WISH images of the indicated genes for 6 hpf control and *adnpa* overexpressing embryos. Note the slightly expanded expression of *chd* and *tbx6* domains, and the slightly reduced expression of *eve1* domain in *adnpa* overexpressing embryos. Dorsal to the left. The vertical black bars are used to compare the expression domain of *tbx6* in Adnp overexpressing and control embryos. **i** Representative morphology of the shield-stage control, *adnpa adnpb* double mutant and Adnpa overexpressing embryos. Black arrows pointing to the dorsal organizer. Dorsal is to the right. All experiments were repeated at least two times. Similar results were obtained and shown are representative images. Scale bars: 50 μm in a-e, 100 μm in f-i.

Table 1 The primers for RT-qPCR analysis

**Mouse genes***	**Forward (5'-3')**	**Reverse (5'-3')**
β-actin	AGAGGGAAATCGTGCGTGAC	CAATAGTGATGACCTGGCCGT
Nanog	ACCCAACTTGGAACAACCAG	CGTAAGGCTGCAGAAAGTCC
Pou5f1	CGTTCTCTTTGGAAAGGTGTTC	GAACCATACTCGAACCACATCC
Klf4	AAGAGGGGAAGAAGGTCGTG	GGTAGTGCCTGGTCAGTTCA
Sox2	ATGGCCCAGCACTACCAG	CCTCCCAATTCCCTTGTATC
Gsc	GCACCATCTTCACCGATGAG	AGGAGGATCGCTTCTGTCGT
T	CTGGGAGCTCAGTTCTTTCG	CCCCTTCATACATCGGAGAA
Gata4	TCTCACTATGGGCACAGCAG	GCGATGTCTGAGTGACAGGA
Gata6	CAAAAGCTTGCTCCGGTAAC	TGAGGTGGTCGCTTGTGTAG
Sox17	GCTTCTCTGCCAAGGTCAAC	CTCGGGGATGTAAAGGTGAA
Foxa2	CCCCTACGCCAACATGAACT	AAGGAGAGAGAGTGGCGGAT
Sox7	AAGTCATGGAAGGCGCTGAC	GGCGCTTGCCTTGTTTCTTC
Adnp	ACGAAAAATCAGGACTATCGG	GGACATTCCGGAAAGACTTT
Sox1	AGACTTCGAGCCGACAAGAG	AACTGTGCAAACAGGTGCAG
Pax6	AGTGAATGGGCGGAGTTATG	ACTTGGACGGGAACTGACAC
Nestin	CCCTGAAGTCGAGGAGCTG	CCCTGAAGTCGAGGAGCTG
Pax2	AAGCCCGGAGTGATTGGTG	CAGGCGAACATAGTCGGGTT
Olig2	TCCCCAGAACCCGATGATCTT	CGTGGACGAGGACACAGTC
Foxd3	CTCTGATCCTGGTCCATCTGTCCT	GGTGCATTTTTGGAAATTCGGTTA
Nptx2	CTCATCGAGTGGGGCAACAA	GCTCACCCACAAATGCTTGG
Tubb3	TAGACCCCAGCGGCAACTAT	GTTCCAGGTTCCAAGTCCACC
Gfap	GAAAACCGCATCACCATTC	TTGTGACTTTTTGGCCTTCC
Ccnd1	CAGAGGCGGATGAGAACAAGT	GCGGTAGCAGGAGAGGAAG
Axin2	CTCCCCACCTTGAATGAAGA	TGGCTGGTGCAAAGACATAG
Myc	GTTGGAAGAGCCGTGTGTG	CGCTGATGTTGGGTCAGTC
Ctnnb1	AGCCGAGATGGCCCAGAAT	AAGGGCAAGGTTTCGAATCAA
Lef1	AGCACGGAAAGAGAGACAGC	GCTGTCATTCTGGGACCTGT
Dkk1	GAGGAAGGCATCATTGAAAAC	CTGTGGCGCAGTCTGATGATC

*: gene names are shown in italic format

Table 2 The primers used for cDNA cloning

Mouse genes*	Forward (5'-3')	Reverse (5'-3')
Adnp	ATGTTCCAACTTCCTGTCAACAATC	GATGCAACACGGCCCATATGC
Adnp-Nter(1-685)	ATGTTCCAACTTCCTGTCAACAATC	TCAGTGGACTAGATGCAGAGTGAT
Adnp-Nter(1-244)	ATGTTCCAACTTCCTGTCAACAATC	TCATTCATGGTCCTCAATGACATGCT
Adnp (245-491)	ATGGAACGGATAGGCTATCAGGTC	TCAGAGGCATTTGCTAGTAAAATTGTG
Adnp (484-685)	ATGCACAATTTTACTAGCAAATGCCTC	TCAGTGGACTAGATGCAGAGTGAT
Adnp-Cter	ATGGTCCGCGACTGTGAAAAGTAC	GATGCAACACGGCCCATATGC
β-catenin	ATGGCTACTCAAGCTGACCTG	TTAAACCTTATCGTCGTCATCCTT
β-catenin (1-55)	ATGGCTACTCAAGCTGACCTG	TCATTCTTCCTCAGGGTTGCCCTT
β-catenin (151-666)	ATGCGTGCAATTCCTGAGCTGACA	TCACTTGTCCTCAGACATTCGGAA
β-catenin (151-320)	ATGCGTGCAATTCCTGAGCTGACA	TCATCCACCACTGGCCAGAATGAT
β-catenin (320-666)	ATCATTCTGGCCAGTGGTGGA TGA	TCACTTGTCCTCAGACATTCGGAA

Table 3 The primers for making WISH probes in zebrafish

Zebrafish genes*	Forward (5'-3')	Reverse (5'-3')
adnpa	CTTTGGAGTCCATGGTGCTG	TAATACGACTCACTATAGGGCCTTTGGGAGTCTGGCAAAC
adnpb	ATGTACCCATACGATGTTCCAGATTACGCTGAATGTTTCAACTCCCAGTGAACAAC	TCCAATCGACAAGGACTGTGTAG
phox2a	CCGGACATCTACACGAGAGAG	TAATACGACTCACTATAGGGGGGAATTTGATGACCACGCTG
neurod1	CGAGCAGAGCCAGGAGAT	TAATACGACTCACTATAGGGAGGGTGGTGTCAAAGAACG
dlx5a	ATGACTGGAGTATTCGACAGA	TAATACGACTCACTATAGGGTCAGTACAACGTTCCTGATC
