# Supplementary material for: ADNP promotes neural differentiation by modulating Wnt/β-catenin signaling
Source: Nat Commun. 2020 Jun 12;11:2984. doi: 10.1038/s41467-020-16799-0 (PMC7293280; doi:10.1038/s41467-020-16799-0)
Supplement: Supplementary file 3 — Reporting Summary [file 41467_2020_16799_MOESM3_ESM.pdf]

nature research

Corresponding author(s): yuhua Sun

Last updated by author(s): 2010-5-12

Reporting Summary

Nature Research wishes to improve the reproducibility of the work that we publish. This form provides structure for consistency and transparency in reporting. For further information on Nature Research policies, see Authors & Referees and the Editorial Policy Checklist.

Statistics

For all statistical analyses, confirm that the following items are present in the figure legend, table legend, main text, or Methods section.

n/a	Confirmed	
☐	☒	The exact sample size (*n*) for each experimental group/condition, given as a discrete number and unit of measurement
☐	☒	A statement on whether measurements were taken from distinct samples or whether the same sample was measured repeatedly
☐	☒	The statistical test(s) used AND whether they are one- or two-sided<br>*Only common tests should be described solely by name; describe more complex techniques in the Methods section.*
☒	☐	A description of all covariates tested
☒	☐	A description of any assumptions or corrections, such as tests of normality and adjustment for multiple comparisons
☐	☒	A full description of the statistical parameters including central tendency (e.g. means) or other basic estimates (e.g. regression coefficient) AND variation (e.g. standard deviation) or associated estimates of uncertainty (e.g. confidence intervals)
☐	☒	For null hypothesis testing, the test statistic (e.g. F, t, r) with confidence intervals, effect sizes, degrees of freedom and P value noted<br>*Give P values as exact values whenever suitable.*
☒	☐	For Bayesian analysis, information on the choice of priors and Markov chain Monte Carlo settings
☒	☐	For hierarchical and complex designs, identification of the appropriate level for tests and full reporting of outcomes
☒	☐	Estimates of effect sizes (e.g. Cohen's *d*, Pearson's *r*), indicating how they were calculated

Our web collection on statistics for biologists contains articles on many of the points above.

Software and code

Policy information about availability of computer code

Data collection	No software used to collect data. Conversion to fastq format of RNA-seq was performed by BGI, Shenzhen China.
Data analysis	Data were analyzed using Student's t-test analysis. Error bars represent s.e.m. RNA-seq data analysis was done by BGI, Shenzhen China. DEGs were defined by FDR < 0.05 and a Log2 fold change >1 fold. Image J software (version 1.45) was used for analysis of WB and IF data. Analysis of FACs data was done by FlowJo 10.5.0 (TreeStar, OR, USA).

For manuscripts utilizing custom algorithms or software that are central to the research but not yet described in published literature, software must be made available to editors/reviewers. We strongly encourage code deposition in a community repository (e.g. GitHub). See the Nature Research guidelines for submitting code & software for further information.

Data

Policy information about availability of data

All manuscripts must include a data availability statement. This statement should provide the following information, where applicable:

- Accession codes, unique identifiers, or web links for publicly available datasets
- A list of figures that have associated raw data
- A description of any restrictions on data availability

RNA-seq and IP-followed by mass spectrometry data are available at https://bigd.big.ac.cn/. The accession numbers are: CRA002148 and CRA001624 (day 0 or ESC state), and CRA001901 (day 3 and day 6 of ESC neural differentiation). All other data are available upon request.

Field-specific reporting

Please select the one below that is the best fit for your research. If you are not sure, read the appropriate sections before making your selection.

☒ Life sciences ☐ Behavioural & social sciences ☐ Ecological, evolutionary & environmental sciences

For a reference copy of the document with all sections, see nature.com/documents/nr-reporting-summary-flat.pdf

Life sciences study design

All studies must disclose on these points even when the disclosure is negative.

Sample size	All experiments were conducted with at least two independent biological replicate cell lines and multiple biological biological replicates. For full list of cell lines used in this study see supplementary information. Sample sizes were determined based on our previous experience and similar studies of other groups. Zebrafish experiments (IF,WISH, overexpression experiments) were performed at least two times. Sample size for zebrafish work (embryos numbers) were determined to satisfy the significance or statistical requirement, similar to other groups in zebrafish community. It has been our routine practice to use more than 20 embryos for IF and WISH experiments, from at least two experimental repeats.
Data exclusions	No data exclusion was done.
Replication	All experiments were performed for al least two times. Q-PCR and rescue were performed al least three times to satisfy significance analysis. Data presented are either representative of of replicates experiments of similar finding or average of replicates as indicated. Reported results were repeated and confirmed for at least two independent experiments. Key experimental findings were reproduced by two investigators involved in the lab.
Randomization	Samples were allocated into experimental groups according genotype or transgenic cell lines. For zebrafish, siblings were used for controls when performing WISH and IF experiments.
Blinding	The investigators were blinded to group allocation during data production, but were not blinded to group allocation during data analysis, for stem cell work described in the manuscript. For WISH and IF experiments of zebrafish embryos, two students independently performed the experiments to ensure the reproducible results. The investigators for zebrafish work were blinded to group allocation.

Reporting for specific materials, systems and methods

We require information from authors about some types of materials, experimental systems and methods used in many studies. Here, indicate whether each material, system or method listed is relevant to your study. If you are not sure if a list item applies to your research, read the appropriate section before selecting a response.

Materials & experimental systems

n/a	Involved in the study	
☐	☒	Antibodies
☐	☒	Eukaryotic cell lines
☒	☐	Palaeontology
☐	☒	Animals and other organisms
☒	☐	Human research participants
☒	☐	Clinical data

Methods

n/a	Involved in the study	
☒	☐	ChIP-seq
☐	☒	Flow cytometry
☒	☐	MRI-based neuroimaging

Antibodies

Antibodies used	ADNP (AF5919, R&D Systems), non-phospho (Active) β-Catenin (Cell Signaling Technology CST, #8814), Phospho-β-Catenin (Ser33/37/Thr41) (CST, #9561), total β-Catenin (CST, #9562), anti-NESTIN (Abcam, ab7659), anti-PAX6 (Proteintech, 12323-1-AP), anti-PCNA (Cusabio, CSB-PA01567A0Rb), anti-GSK3β (Proteintech, 2204-1-AP), anti-pGSK3β (CST, 9336S), anti-PAX2 (Abcam, ab79389), anti-TuJ1 (Abcam, ab78078), anti-GFAP (Abcam, ab53554), anti-HuC/D (Life Technologies, A21271), anti-β-Catenin (Sigma, C7207), Anti-FLAG (F3165, Sigma, 1:1000), anti-MYC antibody (Transgen Biotech, HT101), anti-HA (Abbkine, A02040, 1:1000); HRP-conjugated goat anti-rabbit IgG (GtxRb-003-DHRPX, ImmunoReagents), HRP-linked anti-mouse IgG (7076S, Cell Signaling Technology), HRP-conjugated Affinipure Rabbit Anti-Goat IgG(H+L) (Proteintech,SA00001-4) and Rabbit Control IgG - ChIP Grade (ab46540)
Validation	For ESC work, antibodies used are chosen based on the published work in the field. And each antibody was validated for Western blot - correct size of the detected bands based on the protein marker, as shown in the figures of this manuscript; For IP experiments, antibodies were first tested by WB or pull-down followed WB. For zebrafish work, the FLAG, HuC and beta-catenin antibodies are the most used antibodies for IF and IP in the zebrafish community. The expression pattern has been known. Proper controls were also used for validation.

Eukaryotic cell lines

Policy information about cell lines

Cell line source(s)	mouse ESC R1 line was purchased from Shanghai stem cell bank. Transgenic stem cell lines (FLAG-ADNP Adnp-/- ESCs; Tet-Express inducible FLAG-ADNP Adnp-/- ESCs; inducible HA-beta-Catenin ESCs) shown in the manuscript were constructed in the lab. The human embryonic kidney cell line HEK293T was purchased from Shanghai cell bank (CRL11268).
Authentication	Genotyping followed by Sanger sequencing, followed by qPCR or Western blot
Mycoplasma contamination	all cells lines used in this study were tested negative of Mycoplasma contamination
Commonly misidentified lines (See ICLAC register)	no

Animals and other organisms

Policy information about studies involving animals; ARRIVE guidelines recommended for reporting animal research

Laboratory animals	zebrafish AB line, females and males of 5 month old were used to cross
Wild animals	no use of wild animals
Field-collected samples	no use
Ethics oversight	Institute of hydrobiology

Note that full information on the approval of the study protocol must also be provided in the manuscript.

Flow Cytometry

Plots

Confirm that:

☒ The axis labels state the marker and fluorochrome used (e.g. CD4-FITC).

☒ The axis scales are clearly visible. Include numbers along axes only for bottom left plot of group (a 'group' is an analysis of identical markers).

☒ All plots are contour plots with outliers or pseudocolor plots.

☒ A numerical value for number of cells or percentage (with statistics) is provided.

Methodology

Sample preparation	Neurospheres cell aggregates were washed with DPBS. The aggregates were dissociated into single cells with 0.25% trypsin (BI, 03-050-1A). After fixing with 4% paraformaldehyde at room temperature for 10 minutes, the cells were washed with 0.5% PBSA (0.5% BSA in PBS) and treated with 90% cold methanol for 15 minutes. After extensive washes, the cells were suspended with 200 µl PBSA and filtered with the flow tube. Then the cells were incubated with antibodies at room temperature for 15 minutes. After three-times wash with 0.5% PBSA, the cells were incubated with secondary antibodies (1: 500 dilution in PBSA, AlexaFluor 488) at room temperature for 15 minutes in the dark. The cells were washed with PBSA for two-times and resuspended with 400 µl PBSA, and were analyzed by the BD AccuriC6 flow cytometer.
Instrument	BD AccuriC6 flow cytometer
Software	FlowJo 10.5.0 (TreeStar, OR, USA)
Cell population abundance	Abundance and purity in the relevant cell populations within post-sort fractions were assessed by flow cytometry analysis.
Gating strategy	The gating strategies used in the study have been described in the supplementary figure 2. Negative controls (isotype match antibodies or negative cells) were used to determines negative and positive boundaries.

☒ Tick this box to confirm that a figure exemplifying the gating strategy is provided in the Supplementary Information.
