# Supplementary material for: ADNP promotes neural differentiation by modulating Wnt/β-catenin signaling
Source: Nat Commun. 2020 Jun 12;11:2984. doi: 10.1038/s41467-020-16799-0 (PMC7293280; doi:10.1038/s41467-020-16799-0)
Supplement: Supplementary file 2 — peer review [file 41467_2020_16799_MOESM2_ESM.docx]

Peer Review Information

**Journal:** Nature Communications

**Manuscript title:** ADNP promotes neural differentiation by modulating Wnt/β-catenin signaling

**Corresponding author name(s):** Dr Yuhua Sun

**Editorial notes:**

**Transferred manuscripts**

This manuscript has been previously reviewed at another journal that is not operating a transparent peer review scheme. This document only contains reviewer comments and rebuttal letters for versions considered at *Nature Communications*.

**Transferred manuscripts (no peer review at *Nature Communications*)**

This manuscript has been previously reviewed at another journal that is not operating a transparent peer review scheme. The manuscript was considered suitable for publication without further review at *Nature Communications*.

**Redactions – transferred manuscripts (mention of previous referee reports from elsewhere)**

This manuscript has been previously reviewed at another journal that is not operating a transparent peer review scheme. This document only contains reviewer comments and rebuttal letters for versions considered at *Nature Communications*. Mentions of prior referee reports have been redacted.

**Redactions – transferred manuscripts (mention of the other journal)**

This manuscript has been previously reviewed at another journal that is not operating a transparent peer review scheme. This document only contains reviewer comments and rebuttal letters for versions considered at *Nature Communications*. Mentions of the other journal have been redacted.

**Redactions – unpublished data**

Parts of this Peer Review File have been redacted as indicated to maintain the confidentiality of unpublished data.

**Redactions – confidential patient information**

Parts of this Peer Review File have been redacted as indicated to maintain patient confidentiality.

**Redactions – published data**

Parts of this Peer Review File have been redacted as indicated to remove third-party material where no permission to publish could be obtained.

**Amendments – published data**

[Figure X/The figure on page X] in this Peer Review File has been amended to remove third-party material where no permission to publish could be obtained.

**Published data (credit line for image)**

[Figure X/The figure on page X] in this Peer Review File is reproduced with permission from [Copyright Holder].

**Redactions – reviewer opt-out**

Parts of this Peer Review File have been redacted as indicated as we could not obtain permission to publish the reports of reviewer ###.

**Reviewer comments in marked-up manuscript**

In their review of the [first/second/third/...] version of this manuscript, reviewer ### added their comments to the manuscript file. These comments, excluding minor textual revisions, have been copied into this Peer Review File.

**Reviewer comments & decisions:**

**Reviewer comments, first version:**

Reviewers' comments:

Reviewer #1 (Remarks to the Author):

The article entitled - ADNP promotes neural differentiation by modulating Wnt/β-catenin signaling brings new aspects to in depth research on ADNP. The work is nicely carried out. My suggestions for improvement include minor modifications.
I suggest the authors make sure to properly cite and discuss in more depth the articles below. These articles directly relate to the subject of the current manuscript as follows:

1] ADNP differential nucleus/cytoplasm localization in neurons suggests multiple roles in neuronal differentiation and maintenance.
Mandel S, Spivak-Pohis I, Gozes I.
J Mol Neurosci. 2008 Jun;35(2):127-41. doi: 10.1007/s12031-007-9013-y. Epub 2008 Feb 20.

2] Activity-dependent neuroprotective protein (ADNP) differentially interacts with chromatin to regulate genes essential for embryogenesis.
Mandel S, Rechavi G, Gozes I.
Dev Biol. 2007 Mar 15;303(2):814-24. Epub 2006 Dec 1.

3] NAP Protects against Tau Hyperphosphorylation Through GSK3.
Ivashko-Pachima Y, Gozes I.
Curr Pharm Des. 2018;24(33):3868-3877.


Lastly, although cited – the article below needs to be further discussed:

Novel evolutionary-conserved role for the activity-dependent neuroprotective protein (ADNP) family that is important for erythropoiesis.
Dresner E, Malishkevich A, Arviv C, Leibman Barak S, Alon S, Ofir R, Gothilf Y, Gozes I.
J Biol Chem. 2012 Nov 23;287(48):40173-85. doi: 10.1074/jbc.M112.387027. Epub 2012 Oct 15.

Finally, the authors may also want to refer to the Helsmoortel-Van der Aa syndrome as also known as the ADNP syndrome - e.g. https://www.orpha.net/consor/cgi-bin/OC_Exp.php?Lng=EN&Expert=404448


Reviewer #2 (Remarks to the Author):

In this manuscript, the authors investigate the role of ADNP in regulating neurodevelopment. They provided extensive data to show ADNP promotes neural induction and neural differentiation by stabilizing b-catenin hence enhancing Wnt signaling. The question is important and the finding is potentially interesting and significant. The manuscript contains a large amount of data. However not all the data are providing support for the hypothesis. More importantly, quantitative analyses are either lacking or inappropriate.

Main concerns
(1) ADNP null mouse study was published in 2003. Therefore the major novelty and discovery by current authors is that ADNP stabilizes b-catenin therefore enhances Wnt signaling during ESC neural induction and differentiation. The figures 1-7 systematically unveil this main point by showing phenotypes of ADNP-different ESCs and by identifying ADNP binding to b catenin in and prevent the formation of degradation complex. The authors should have focused on these analyses. However it is unclear why cerebellar neuron differentiation data are added (Fig 8)-even though IHC of 10-weeks old adult mice show relatively higher levels of ADNP in cerebellar granule neurons, it does not mean the development of these neurons are regulated by ADNP. Does Helsmoortel-Van der Aa syndrome patients have cerebellar deficits implying developmental defect of cerebellum? Then the authors added neuroprogenitor proliferation and apoptosis data (Fig9). If N neuroprogenitor phenotypes are part of ESC neural induction and differentiation phenotypes, this figure should be at the beginning of this study and the proliferation/cell death mechanism should be assessed within figures 1-7. It is even more puzzling why including zebra fish data (Fig 10). What can this figure add to what we have already known from the study of ADNP mouse mutant? The figures 8-10 do not provide support for the main conclusion of the paper.
(2) I have extensive concerns with quantitative analysis of this manuscript. First, western blotting was used extensively to support the claim and draw conclusion but there is NO quantification of any western blots starting from Fig 1C and throughout the paper. Second, even some qPCR data (e.g. 1H, 2C, S3b) have neither error bar nor statistical analysis. Third, none of the differentiation analysis have quantitative data. Fourth, cell proliferation was determined by number of PH3 cells (Fig 9a), which should be % cells that are PH3+. The cell pellet size (Fig 9a) is not a precise measurement for cell number.
(3) Some important data seem to be missing. Page 4 they performed RNA-seq of mutant and control ESCs “Our RNA-seq analysis showed that the primitive endoderm genes such as Gata6, Gata4, Sox17, Sox7 and Sparc were slightly up-regulated in the absence of ADNP.” Where is RNA-seq data? The cerebellar differentiation analysis did not provide any validation showing the differentiated cells are the correct cell types (figure 8)

(4) The language is not precise and even mistaken. For example,
Page 15: “loss of ADNP failed to induce β-Catenin degradation, reflecting that ADNP-regulated β-Catenin degradation was dependent on the ubiquitin-proteasome pathway (Fig. 6e)”. Protein degradation can be mediated by other pathways, the conclusion of ubiquitin pathway has no support.
Page 19. “To explore this, vector slices from ten-week-old mouse embryonic brains were subjected to immunohistochemistry”. These are not embryonic brains.
Page 19 “This observation suggested that ADNP is cell-autonomously required for mouse embryonic brain development and maturation, especially for the cerebellum.” This is a strange conclusion. First, this IHC does not provide any data to support “cell autonomous” regulation. Second, the expression pattern in adult cerebellum does not provide strong support for developmental requirement.

Specific comments:
(1) Figure 1. Creation of CRISPR gene mutation for Adnp-/- ESCs: off target effects should be analyzed.
(2) Figure 2g: there seem to be a lot of double labeling of TuJ1 and GFAP. The number and % marker positive cells should be quantified.
(3) Figure 9 shows both reduction in proliferating cells and increased active Caspase 3 levels. However neither analysis was done in correct quantitative method, it is unclear whether reduced cell number is due to either or both.


Reviewer #3 (Remarks to the Author):

This interesting manuscript uncovers a novel role for ADNP (Activity Dependent Neuroprotective Protein) as an important player and modulator of the Wnt pathway. They demonstrate that ADNP interacts with b-Catenin, and that this interaction leads to its active function/and or protection from degradation, and show that KD or KO of ADNP leads to reduced Wnt signaling. They explore the function of ADNP in the context of neural development using mouse embryonic setm cells, and zebra fish embryos. They provide a series of loss of function, rescue and gain of function experiments that demonstrate that ADNP is required for neural precursor and neural differentiation, that this role is mediated and can be rescued by b-Catenin signaling. They further show that KO of ADNP leads to a considerable reduction of b-Catenin, and they suggest that the binding alters the levels of cytoplasmic and not nuclear b-Catenin . Characterizing the link between ADNP and WNT signaling they demonstrate that ADNP can bind to b-Catenin, and that the binding is mainly mediated by the armadillo domains of b-Catenin.

To test the involvement of ADNP in developmental deficits of the cerebellum, the authors attempt to assess cerebellar neuron differentiation in Wt control and ADNP KO cells (figure 8). It is hard to understand the logic of this experiment given that they have documented the effects of the removal of ADNP for neuronal precursors and neural ectoderm differentiation. A conditional inducible removal of ADNP would be a more convincing experiment.

On the last series of experiments, regarding analysis of ADNP in zebrafish embryos (fig 10) the
authors find that noticeable phenotypes arise at 36 hpf and later, yet the Wnt analysis suggest that deficits are already appreciable by 8hpf (fig10g and h). The levels of Wnt readout genes or Topflash clearly are lower in the dKO than in wt, but I wonder a) how is this remaining Wnt readout generated, and b) how would it compare to a b-Catenin KO?

A key missing point is an adequate presentation and discussion of the differences and similarities found between this study and that of Ostapcuk, V. et al. 2018 (Reference 4 of this manuscript). For example, that manuscript reports immediate deficits in stemness not found here. And suggests that ADNP might control lineage specific genes in stem cells.

It is also worth mentioning, that if ADNP also functions as a classic TF, the phenotypes observed could be confounded by the multiple roles it plays. If some of these targets are for example, controlled by ADNP and by WNT.

Another intriguing issue is that no earlier WNT phenotypes prior to the ones described are noted in the KO embryos and lines. WNT signaling is thought to be critical during early embryonic development, yet mouse and fish lacking ADNP make it well past gastrulation…Similarly, Wnt signaling is supposed to be required for ES maintenance, and here the ADNP KO lines go on unaltered for several passages. This suggest that the WNT pathway does not rely on ADNP at this stages, perhaps its function is redundant, compensated, or not necessary. But one would expect that if it is required for Wnt/b-Catenin signaling its KO should mimic that of b-Catenin.


Below are a few typos and other minor issues with suggestions:

Line 118: …ADNP is required for the  neuroectodermal differentiation of mESCs  
It seems more appropriate to state that ADNP is required for the expression of neuroectodermal markers.

Line 286: “neurite outgrowth by interacting with tubulin, and protect neurons and glia exposing” should read “exposed”  

Line 323:  “remained lower in the absence of ADNP than its presence” …“than in its presence”

Line 340:  “we concluded that ADNP is required for the stability of β-Catenin in ESCs and during ESC differentiation towards a neural cell fate.” This statement might be to strong as the KO displays lots of nuclear b-Catenin…and KO mice developing for a while…presumably with active b-Catenin signaling, as stated above as a major concern, a more precise statement is required and a discussion/comment that includes these arguments.

line354:  “ As shown in Fig. 6a-g” the figures meant appear to be 6c and g…?
Line 397:  “form neural progenitors and neuronal cells with gene expression profiles similar to that of wild type ESCs (Fig. 6h, 7g)”. The levels are similar but more precisely, “slightly lower”

line 516: what is the neuro-significance of this Tg elavl3?
line 541:  “De novo mutations in ADNP have recently“ should read have been recently

Lines 552 to 555 states: ”These observations suggested that the intra-cellular localization of ADNP is tightly regulated during neural induction, or that ADNP plays an important role in the cytoplasm for the induction, maintenance and mature of neural cell types”. But in reality, these options are not mutually exclusive. It is also curious that it is thought as a transcription factor, and it is found in the nucleus, so in addition to the epigenetic links described, one might still consider that it could modulate directly the expression of some unidentified targets.

Line 571: “exposing to toxins”…exposed to toxins.

Line 578” “we showed that ADNP-NAP alone can interact with β-Catenin, suggesting that NAP  
can execute similar function as ADNP in the regulation of Wnt/β-Catenin signaling pathway.” Given the overexpression and in vitro nature of the experiments for this portion, the statement should be much more cautious.

Line 614: “synaptic function and the chromatin remodeling.”  


Line 621: “This may imply why ADNP is…” This may explain why

**Author rebuttal, first version:**

Reviewer #1 (Remarks to the Author):

The article entitled - ADNP promotes neural differentiation by modulating Wnt/β-catenin signaling brings new aspects to in depth research on ADNP. The work is nicely carried out. My suggestions for improvement include minor modifications.
I suggest the authors make sure to properly cite and discuss in more depth the articles below.

Response: I appologize for the inadequate citation of some important ADNP-related literature. Now the articles below are cited and discussed (cited in referenced literature No. 3, 18 and 23; discussed in line 332-334; 372-375; 613-616).

These articles directly relate to the subject of the current manuscript as follows:

1] ADNP differential nucleus/cytoplasm localization in neurons suggests multiple roles in neuronal differentiation and maintenance.
Mandel S, Spivak-Pohis I, Gozes I.
J Mol Neurosci. 2008 Jun;35(2):127-41. doi: 10.1007/s12031-007-9013-y. Epub 2008 Feb 20.

2] Activity-dependent neuroprotective protein (ADNP) differentially interacts with chromatin to regulate genes essential for embryogenesis.
Mandel S, Rechavi G, Gozes I.
Dev Biol. 2007 Mar 15;303(2):814-24. Epub 2006 Dec 1.

3] NAP Protects against Tau Hyperphosphorylation Through GSK3.
Ivashko-Pachima Y, Gozes I.
Curr Pharm Des. 2018;24(33):3868-3877.


Lastly, although cited – the article below needs to be further discussed:

Novel evolutionary-conserved role for the activity-dependent neuroprotective protein (ADNP) family that is important for erythropoiesis.
Dresner E, Malishkevich A, Arviv C, Leibman Barak S, Alon S, Ofir R, Gothilf Y, Gozes I.
J Biol Chem. 2012 Nov 23;287(48):40173-85. doi: 10.1074/jbc.M112.387027. Epub 2012 Oct 15.
Response: this paper is further discussed in the revised manuscript (line 705-708).

Finally, the authors may also want to refer to the Helsmoortel-Van der Aa syndrome as also known as the ADNP syndrome - e.g. [https://www.orpha.net/consor/cgi-bin/OC_Exp.php?Lng=EN&Expert=404448](https://urldefense.proofpoint.com/v2/url?u=https-3A__www.orpha.net_consor_cgi-2Dbin_OC-5FExp.php-3FLng-3DEN-26Expert-3D404448&d=DwMGaQ&c=vh6FgFnduejNhPPD0fl_yRaSfZy8CWbWnIf4XJhSqx8&r=-6Lu-f4SRMW3ibr_ys1_zi6nDH4vpWIL7d68j4rghbY&m=qmP0q413ojdarudBbMHqPUsgumtAp5PvkvGCOHC3iIM&s=mNTGh4FLY7gfjiIMzduu_q-REy6b-2N1-yXTbyOccBQ&e=)

Response: Information has been added (line 8 in the abstract of the revised manuscript).

Reviewer #2 (Remarks to the Author):

In this manuscript, the authors investigate the role of ADNP in regulating neurodevelopment. They provided extensive data to show ADNP promotes neural induction and neural differentiation by stabilizing b-catenin hence enhancing Wnt signaling. The question is important and the finding is potentially interesting and significant. The manuscript contains a large amount of data. However not all the data are providing support for the hypothesis. More importantly, quantitative analyses are either lacking or inappropriate.

Response: We have generated some new data to support our hypothesis (added figures include Figure S1a; Figure 6e; 6h; Figure 7i; Figure S7; Figure 8 g-h; Figure S8e; Figure 9 and Figure S9). Quantitative analyses have been added in the revised manuscript (Figure 1h; Figure S1a; Figure 2c; Figure S3b; Figure 2h; Figure 4g; Figure S4g; Figure 7h; Figure S7b; line 85-88; 152-155; 163-165; 168-172; 271-273). All quantification for WB was annotated in the figures.

Main concerns
(1) ADNP null mouse study was published in 2003. Therefore the major novelty and discovery by current authors is that ADNP stabilizes b-catenin therefore enhances Wnt signaling during ESC neural induction and differentiation. The figures 1-7 systematically unveil this main point by showing phenotypes of ADNP-different ESCs and by identifying ADNP binding to b catenin in and prevent the formation of degradation complex. The authors should have focused on these analyses.

Response: Thank you for the comments. We have adjusted our manuscript to focus on these parts.

However it is unclear why cerebellar neuron differentiation data are added (Fig 8)-even though IHC of 10-weeks old adult mice show relatively higher levels of ADNP in cerebellar granule neurons, it does not mean the development of these neurons are regulated by ADNP. Does Helsmoortel-Van der Aa syndrome patients have cerebellar deficits implying developmental defect of cerebellum? Then the authors added neuroprogenitor proliferation and apoptosis data (Fig9). If N neuroprogenitor phenotypes are part of ESC neural induction and differentiation phenotypes, this figure should be at the beginning of this study and the proliferation/cell death mechanism should be assessed within figures 1-7.

Response: As the cerebellum dysfunction in some of the Helsmoortel-Van der Aa patients can manifest in early childhood (show different extent of intellectual disability and cognitive deficits), we initially wanted to test whether there is a direct link between loss of ADNP and developmental defects in cerebellar neuron cell types using a ESC model. However, as you have pointed out, 1) even mice show high levels of ADNP in cerebellar granule neurons, it does not mean that the development of these neurons are regulated by ADNP, 2) or the observed late cerebellar neuronal phenotypes could be due to an indirect effect caused by loss of *Adnp*. So, we agree with your comment on the logic of this part. In addition, the neuroprogenitor death phenotype as part of *Adnp-/-* ESC neural induction has been reported by Ostapcuk (2018, Nature). Therefore, to focus on the main finding of this manuscript, we would like to drop Figures 8-9 out and focus on Figures 1-7 and Figures 9-10 according to your suggestions. Now, our new manuscript have been revised to focus on the effects of loss of ADNP on neural induction and early neural differentiation using both ESC neural differentiation and zebrafish models.

It is even more puzzling why including zebra fish data (Fig 10). What can this figure add to what we have already known from the study of ADNP mouse mutant? The figures 8-10 do not provide support for the main conclusion of the paper.

Response: Thanks to the reviewer’ comments, we have revisited the zebrafish part of work and have generated new data. With zebrafish as a good in vivo model, we now show that zAdnp might be a temporal or tissue specific modulator of Wnt signaling pathway which was not addressed in the previous manuscript. Some key data have been provided (Figure 8g-h; Figure S8e; 9a-h and S9a-g, j). we showed that 1) zADNP co-localizes and interacts with β-Catenin in zebrafish embryos (Figure 9a-c; line 480-509); 2) why zAdnp may function as a temporal specific modulator of the Wnt signaling. zAdnp protein is ubiquitously expressed and predominantly localized in nuclei of pre-gastrulating and gastrulating embryos, and predominantly localized in the cytoplasm of embryos after gastrulation (Fig. 9a,b; Supplementary Fig. 9a,b). Loss of Adnpa and Adnpb had a minimal effect on β-Catenin of embryos in pre-gastrulation and gastrulation stages when β-Catenin is critically required for axis formation and proper convergent-extension movement. However, Adnp depletion caused a reduced β-Catenin levels of embryos after gastrulation (Fig. 9d-e; line 510-525). Based on these data, we propose that zAdnp regulates β-Catenin levels predominantly in the cytoplasm during zebrafish neurogenesis, while it has minimal role for the regulation of β-Catenin in pre-gastrulating and gastrulating embryos. Thus, our zebrafish work on one hand shows the functional conservation of ADNP in vertebrate neurodevelopment, and on the other hand provide some new insights into how ADNP functions during embryogenesis: ADNP is not a core component of Wnt signaling, but a temporal specific modulator of Wnt signaling.

(2) I have extensive concerns with quantitative analysis of this manuscript. First, western blotting was used extensively to support the claim and draw conclusion but there is NO quantification of any western blots starting from Fig 1C and throughout the paper.

Response: Quantification has been added for WB, which appears in Figure 1c, 2h, Figure 6, Figure 7 and Figure 9f (annotated in the figures). Using Photoshop we expressed the quantitative data as bar graphs with error bar and significance based on the experimental replicates. Due to the limited space of the Figures, the bar graphs were not included. The quantification method is described at line 861-870 of the revised manuscript.

Second, even some qPCR data (e.g. 1H, 2C, S3b) have neither error bar nor statistical analysis.

Response: these have been corrected and appear in Figure 1e,h; Figure 2c,f; Figure S3b.

Third, none of the differentiation analysis have quantitative data.

Response: the quantitative data have been added. We expressed the quantitative data as bar graphs with error bar and significance based on the experimental replicates (Figure 2h; Figure S2c; S2e; Figure 4g; Figure 7h; Figure S7b). The quantification data were described in the text of the revised manuscript (line 85-88; 152-155; 163-165; 168-172; 271-273). The quantification method is described at line 916-925 of the revised manuscript.

Fourth, cell proliferation was determined by number of PH3 cells (Fig 9a), which should be % cells that are PH3+. The cell pellet size (Fig 9a) is not a precise measurement for cell number.

Response: revised

(3) Some important data seem to be missing. Page 4 they performed RNA-seq of mutant and control ESCs “Our RNA-seq analysis showed that the primitive endoderm genes such as Gata6, Gata4, Sox17, Sox7 and Sparc were slightly up-regulated in the absence of ADNP.” Where is RNA-seq data?

Response: RNA-seq data added (line 85-88; Figure S1a). The original data were deposited at <https://bigd.big.ac.cn/> in Beijing Genomics Institute, under the accession number of CRA002148 (*Adnp* shRNA knockdown)and CRA001624 (*Adnp-/-* ESCs).

The cerebellar differentiation analysis did not provide any validation showing the differentiated cells are the correct cell types (figure 8)
As discussed above, Figure 8 is now removed as suggested.

(4) The language is not precise and even mistaken. For example,
Page 15: “loss of ADNP failed to induce β-Catenin degradation, reflecting that ADNP-regulated β-Catenin degradation was dependent on the ubiquitin-proteasome pathway (Fig. 6e)”. Protein degradation can be mediated by other pathways, the conclusion of ubiquitin pathway has no support.

Response: We have revised this sentence (line 351-354).

Page 19. “To explore this, vector slices from ten-week-old mouse embryonic brains were subjected to immunohistochemistry”. These are not embryonic brains.

Response: this was a mistake. This sentence is removed. Thanks.

Page 19 “This observation suggested that ADNP is cell-autonomously required for mouse embryonic brain development and maturation, especially for the cerebellum.” This is a strange conclusion. First, this IHC does not provide any data to support “cell autonomous” regulation. Second, the expression pattern in adult cerebellum does not provide strong support for developmental requirement.
Response: This sentence was written without supporting experimental evidence, and should be avoided. As part of the ESC cerebellum differentiation, this sentence has been removed.

Specific comments:
(1) Figure 1. Creation of CRISPR gene mutation for Adnp-/- ESCs: off target effects should be analyzed.

Response: We have generated additional two *Adnp* mutant alleles (see below. up panel), and the resulting mutant ESCs could be passaged for many generations, exhibiting similar gene expression (middle) and morphological aspects (bottom panel). Besides, our *Adnp* shRNA knockdown ESCs also resemble mutant ESCs as stated in the text (line 104-107; Figure S1a-f). Thus, all mutants and Adnp KD ESCs exhibited similar phenotypes, which was strongly against the off-target effect. Also, the rescue data also supported this (line 95-96; 154-155; 347-349).

(2) Figure 2g: there seem to be a lot of double labeling of TuJ1 and GFAP. The number and % marker positive cells should be quantified.

Response: Quantification has been added. Please find it in the revised manuscript (Figure S2c; line 164-165; 168-171).

(3) Figure 9 shows both reduction in proliferating cells and increased active Caspase 3 levels. However neither analysis was done in correct quantitative method, it is unclear whether reduced cell number is due to either or both.
Response: As you have pointed out, this part add little to the main conclusion of this manuscript, we would like to leave this part out. Also, cell death phenotype has been reported by Ostapcuk (2018).


Reviewer #3 (Remarks to the Author):

This interesting manuscript uncovers a novel role for ADNP (Activity Dependent Neuroprotective Protein) as an important player and modulator of the Wnt pathway. They demonstrate that ADNP interacts with b-Catenin, and that this interaction leads to its active function/and or protection from degradation, and show that KD or KO of ADNP leads to reduced Wnt signaling. They explore the function of ADNP in the context of neural development using mouse embryonic setm cells, and zebra fish embryos. They provide a series of loss of function, rescue and gain of function experiments that demonstrate that ADNP is required for neural precursor and neural differentiation, that this role is mediated and can be rescued by b-Catenin signaling. They further show that KO of ADNP leads to a considerable reduction of b-Catenin, and they suggest that the binding alters the levels of cytoplasmic and not nuclear b-Catenin. Characterizing the link between ADNP and WNT signaling they demonstrate that ADNP can bind to b-Catenin, and that the binding is mainly mediated by the armadillo domains of b-Catenin.

To test the involvement of ADNP in developmental deficits of the cerebellum, the authors attempt to assess cerebellar neuron differentiation in Wt control and ADNP KO cells (figure 8). It is hard to understand the logic of this experiment given that they have documented the effects of the removal of ADNP for neuronal precursors and neural ectoderm differentiation. A conditional inducible removal of ADNP would be a more convincing experiment.
Response: We thank the reviewer for the comments. As the cerebellum dysfunction in some of the Helsmoortel-Van der Aa patients can manifest in early childhood (exhibit different extent of intellectual disability and cognitive deficits), we initially wanted to test whether there is a direct link between loss of ADNP and developmental defects in cerebellar neuron cell types. However, as you and reviewer 2 both have pointed out, even mice show high levels of ADNP in cerebellar neurons, it does not mean that the development of these late-developing neurons is directly regulated by ADNP. Due to the flawed logic, in our revised manuscript, the cerebellar neuron differentiation part is removed (figure 8). Now, with our new data incorporated, this revised manuscript have been revised to focus on the effects of loss of ADNP on neural induction and early neural differentiation (a direct effect caused by loss of ADNP).

On the last series of experiments, regarding analysis of ADNP in zebrafish embryos (fig. 10) the authors find that noticeable phenotypes arise at 36 hpf and later, yet the Wnt analysis suggest that deficits are already appreciable by 8hpf (fig10g and h). The levels of Wnt readout genes or Topflash clearly are lower in the dKO than in wt, but I wonder a) how is this remaining Wnt readout generated, and b) how would it compare to a b-Catenin KO?

Response: we appreciate the reviewer’ comments. In the former manuscript, we reported that in the absence of zAdnp, the expression of Wnt readout genes was somewhat reduced, and Topflash activities decreased in late gastrulating zebrafish embryos. *adnpa* and *adnpb* double mutant embryos at 24 hpf exhibit mild axis formation phenotypes (Supplementary Figure 8d). Thus, double mutant embryos exhibited mild DV patterning and Wnt-related phenotypes, but overall they do not look like embryos with reduced Wnt/beta-catenin signaling, particular to loss of function b-Catenin embryos which exhibited severe ventralized phenotype (Kelly et al., 2000, Development; Wu et al., 2012, PloS Biology). This observation also suggested that there was remaining Wnt signaling in the 8 hpf double knockout embryos.

Inspired by your comments, we speculated that Adnp might not be a core Wnt signaling component in pre-gastrulating and gastrulating embryos but function as a modulator. To address the question, we carefully compared the morphology of shield-stage control, Adnp overexpressing, and double mutant embryos (Figure below, dorsal to the right). It can be seen that Adnp overexpression indeed causes convergent-extension (CE) defects and enlarged dorsal organizer, while Adnp depletion leads to a slightly reduced dorsal organizer (CE looks fine).

Consistently, Adnpa overexpressing embryos showed CE problem and dorsalized phenotype at 24 hpf (see below) and 48 hpf (Supplementary Figure 9h) which is similar to that of β-Catenin overexpressing embryos (Wu et al., 2012, PloS Biology), while a small portion of double mutant embryos are ventralized (Supplementary Figure 8d).

Nevertheless, in 16 and 24 hpf mutant embryos, a reduction of β-Catenin levels were indeed observed (Fig. 9c-e). Thus, loss of Adnp had a significant role after gastrulation. Together, these observations suggest that Adnp manipulation does cause Wnt related phenotypes but only Adnp overexpressing has a significant effect. They also suggested that Adnp might not be a core component of Wnt signaling pathway in embryos at pre-gastrulation and gastrulation stages as loss of Adnp has minimal effect on beta-Catenin levels and causes DV patterning problem. In stead, Adnp might be a temporal or tissue specific modulator of Wnt signaling (Adnp functions to modulate Wnt signaling after gastrulation). This may explain why there are remaining Wnt readout.

In summary, thanks to your comments, we have revisited the role of ADNP in the regulation of Wnt/beta-catenin signaling, and have made some important corrections for the previous statement. Now using zebrafish as in vivo model we demonstrate that ADNP might function as a temporal or tissue specific modulator of the Wnt signaling pathway. Please see all the details in the revised manuscript.

A key missing point is an adequate presentation and discussion of the differences and similarities found between this study and that of Ostapcuk, V. et al. 2018 (Reference 4 of this manuscript). For example, that manuscript reports immediate deficits in stemness not found here.
Response: Ostapcuk et al. 2018 reported that loss of Adnp leads to an immediate defects in ESC stemness, as evidenced by the abnormal morphology, reduced alkaline phosphotase staining, and deregualation of lineage-specifying genes. In our hand, however, *Adnp-/-* ESCs exhibit a milder phenotype compared to the counterpart ESCs by Ostapcuk. First, the newly established *Adnp-/-* ESCs exhibited a ESC-like morphology and strong alkaline phosphatase activities (Fig. 1d; Figure S1c). They could be passaged for many generations in the LIF/KSR medium. Only prolonged depletion of ADNP leads to loss of ESC phenotype (line 93-99). Second, the expression of pluripotency-related genes such as *Pou5f1* and *Nanog* was barely changed in our newly established *Adnp-/-* ESCs (Fig. 1e; also in our RNA-seq data), while the expression of these genes was significantly reduced in mutant ESCs by Ostapcuk et al. Third, the lineage-specifying genes and other developmental genes were deregulated to a much lower extent in our mutant ESCs compared to that by Ostapcuk. For instances, the up-regulation of key PrE genes such as *Gata4*, *Gata6* and *Sox17* was within 2-3 fold range, while this was over 5 times more in mutant cells by Ostapcuk (Fig. 1e; also in our RNA-seq data under No. CRA002148 and CRA001624 in BGI database). We think that the discrepancy might be due to the nature of the *Adnp* mutant alleles that generated by Ostapcuk and our group. In Ostapcuk’s work, a very large fragment of *Adnp* gene (including exon 3 and 4, the most part of exon 5 as well as intron 3 and 4) was deleted. In that case, it is possible that the deleted large fragment of *Adnp* gene contains cis-elements bearing some important functions, such as involving enhancer or non-coding RNA functions. In our work, only 4 or 5 bp deletion in exon 4 of *Adnp* gene was introduced, which should only disrupt *Adnp* gene function. Importantly, we showed that FLAG-tagged ADNP could largely rescue the phenotypes of *Adnp-/-* ESCs which was not reported by Ostapcuk (Fig. S1c; line 94-96).

All the above could be found at line 559-590 of the revised manuscript.

And suggests that ADNP might control lineage specific genes in stem cells.

To answer the question “ADNP might control lineage specific genes in stem cells”. ADNP contains nine zinc fingers, a homeobox domain and a PxLxP motif, suggesting that it functions as a transcription factor (Pinhasov et al., 2003). Although the majority of ADNP bound sites are associated with protein-coding genes, most ADNP ChIP-seq peaks are not found directly at gene promoter regions (Kaaij et al., 2019, Cell). In addition, many genes strongly bound by ADNP are not deregulated in the absence of ADNP. Of note, ADNP barely binds to lineage-specifying genes, including key mesodermal genes such as *Gsc* and *T,* neuroectodermal regulatory genes such as *Pax6*, *Pax2*, *Olig2* and *Sox1,* as well as endodermal regulatory genes such as *Sox17,* *Sox7* and *Gata6* (our unpublished data, see below).

These observations suggested that ADNP may not be involved in directly controlling these genes as a classic transcription factor in ESCs. Considering that ADNP associates with chromatin remodelers such as CHD4 and BRG1 and competes with the master chromatin architcture protein CTCF, we speculated that it might regulate gene expression by both indirect mechanisms (such as epigenetic mechanism or modulating Wnt signaling pathway in this work) and direct mechanism (as a classic transcription factor).

In ESCs, loss of ADNP had a minimal effect on the expression of neuroectodermal regulatory genes such as *Pax6*, *Olig2* and *Sox1,* as well as Wnt-related genes (fold change < 2; RPKM <2; Figure 1e; line 90-91; also see our RNA-seq data under No. CRA002148 and CRA001624 in BGI database). Therefore, we believe that the change in the expression of neural genes during ESC neural differentiation was not due to the expression change of these genes in *Adnp-/-* ESCs. In fact, we are preparing another manuscript which specifically addressed the question.

As the next question is closely related to this question, please see more details below.

It is also worth mentioning, that if ADNP also functions as a classic TF, the phenotypes observed could be confounded by the multiple roles it plays. If some of these targets are for example, controlled by ADNP and by WNT.

Response: we thank the reviewer for the comment. As stated above, although the majority of ADNP bound sites are associated with protein-coding genes, most ADNP ChIP-seq peaks are not found directly at gene promoter regions (Kaaij et al., 2019, *Cell*: 178, 1437-1451). In addition, many genes heavily bound by ADNP are not deregulated in the absence of ADNP. It seems that gene expression change by loss of ADNP is not predictable by ADNP binding (manuscript in prep). In particular, ADNP ChIP-seq peaks were barely found at key regulatory elements of developmental regulatory genes (see ChIP-seq plot below; grey color: promoter regions; Green color: enhancer regions). These observations suggested that ADNP might not be involved in directly regulating these genes as a classic transcription factor (by binding to gene promoter or enhancer).

Considering that ADNP associates with chromatin remodelers such as CHD4 and BRG1 and competes with the master chromatin architcture protein CTCF (Mandel et al., 2007; Ostapcuk et al., 2018; Kaaij et al., 2019), we speculate that it might control some of genes by indirect mechanism (by modulating ESC chromatin structure or epigenetic landscape) and some of the genes by direct mechanism (as a classic transcription factor). Thus, we can not exclude the possibility that the observed phenotypes in this work might be due to the multiple roles by ADNP, and that some of the genes could be affected by ADNP transcriptional activity and by WNT. We discussed this in the revised manuscript (line 591-608). But it seems that neural genes such as *Pax6* and *Olig2* might not be regulated by the direct mechanism unless ADNP is recruited to these genes during ESC neural differentiation.

Another intriguing issue is that no earlier WNT phenotypes prior to the ones described are noted in the KO embryos and lines. WNT signaling is thought to be critical during early embryonic development, yet mouse and fish lacking ADNP make it well past gastrulation…Similarly, Wnt signaling is supposed to be required for ES maintenance, and here the ADNP KO lines go on unaltered for several passages. This suggest that the WNT pathway does not rely on ADNP at this stages, perhaps its function is redundant, compensated, or not necessary. But one would expect that if it is required for Wnt/b-Catenin signaling its KO should mimic that of b-Catenin.
Response: we appreciate the reviewer for the comments, which let us rethink our manuscript and helped us to greatly improve the current work. To answer why there is no self-renewal phenotype of KO ESCs. First of all, it is generally believed that canonical Wnt signaling has been implicated in mouse and human embryonic stem cell (ESC) maintenance (Sato et al., 2004; Ten Berge et al., 2011). However, the requirement of β-Catenin is still controversial (Kelly et al., 2011). For instances, Lyashenko et al. (2011) found that self-renewal is maintained in the absence of β-Catenin but mesendodermal germ layer formation and neuronal differentiation are defective. Xu et al. (2016, PNAS) showed that inhibition of Wnt/β-Catenin signaling in naive hESCs did not cause differentiation, but induced primed hESC-like profiles. So it is proposed that β-Catenin transactivation function, which is considered to be associated with Wnt signaling, is largely dispensable for ESC self-renewal, but required for differentiation. Second, ADNP is predominantly nuclear localized in ESCs. In this work, we proposed that ADNP regulates β-Catenin levels in the cytoplasm (Figure 6). Consistently, loss of ADNP only had a minimal effect on the β-Catenin levels in ESCs (Figure S6a). Third, we think that ADNP might also function as a temporal or tissue specific modulator of Wnt signaling. Loss of ADNP had greater effect on β-Catenin levels during neural differentiation than in ESC state (Figure 6c-e). Thus, we propose that Wnt signaling is not strictly dependent on ADNP in ESCs, which may explain why there was no immediate phenotypes in *Adnp-/-* ESCs (ADNP KO lines remain undifferentiated for several passages).

To answer the question for the early phenotypes in mutant zebrafish embryos. As discused above, Wnt/beta-Catenin signaling is required to govern the axis formation, and β-Catenin nuclear localization in early embryos (high-stage, 512-cell) is known critical for proper embryonic axis formation. Its depletion or cellular mis-localization caused severe ventralized embryos and overexpressing β-Catenin led to dorsalized embryos (Kelly et al., 2000; Wu et al., 2012; Yan et al., 2018; Yang et al., 2015). We examined the cellular localization of Adnpa in early embryos, and found that zAdnpa is predominantly localized in the nuclei of pre-gastrulating and gastrulating embryos (Figure 9a, b). We found that β-Catenin nuclear localization or its levels in 3.5 and 6 hpf embryos are not significantly altered in the absence of Adnp, which is in line with the that loss of Adnp had minimal effect on axis formation governed by the Wnt/β-Catenin signaling (line 510-517; Figure S9f, g). As Adnp primarily functions to regulate the stability of β-Catenin in cytoplasm, this may explain why loss of Adnp embryos exhibit mild Wnt or β-Catenin phenotypes.

To explain why overexpression of Adnpa can cause dorsalized embryos. Adnp is ubiquitously expressed and predominantly localized in nucleus of pre-gastrulating (shown below, up) and gastrulating embryos (bottom). In 512-cell stage and oblong (3.5 hpf) stages, Adnp and nuclear localized β-Catenin in dorsal blastomere and yolk syncytial layer (YSL) are co-localized. We hypothesized that the interaction of Adnpa and β-Catenin will likely promote the function of β-Catenin in the nucleus. Consistently, when Adnpa was overexpressed, there was a slight expansion of the dorsal marker *chd* and reduction of the ventral marker *eve1*, an increased expression of Wnt target gene *tbx6,* and enlarged dorsal organizer (Figure S9h-j; line 537-547)*.* Thus, Adnp can act in the Wnt pathway early when it is over expressed, explaining why Adnpa overexpression lead to dorsalization of zebrafish embryos.

To summary, based on our new data, we propose that ADNP might function as a temporal or tissue modulator of Wnt/β-Catenin signaling pathway: ADNP has a more significant role in regulating β-Catenin levels (Wnt signaling) after gastrulation and during neurogenesis in zebrafish embryos, and during ESC neural induction and neuronal differentiation.

Below are a few typos and other minor issues with suggestions:
Line 118: …ADNP is required for the neuroectodermal differentiation of mESCs
It seems more appropriate to state that ADNP is required for the expression of neuroectodermal markers.
Response: revised as suggested (now line 125-126).

Line 286: “neurite outgrowth by interacting with tubulin, and protect neurons and glia exposing” should read “exposed”
Response: corrected (now line 307)

Line 323:  “remained lower in the absence of ADNP than its presence” …“than in its presence”
Response: revised (line 347)

Line 340:  “we concluded that ADNP is required for the stability of β-Catenin in ESCs and during ESC differentiation towards a neural cell fate.” This statement might be to strong as the KO displays lots of nuclear b-Catenin…and KO mice developing for a while…presumably with active b-Catenin signaling, as stated above as a major concern, a more precise statement is required and a discussion/comment that includes these arguments.
Response: the statement is revised as “we concluded that ADNP regulates the stability of cytoplasmic β-Catenin in ESCs and during ESC differentiation towards a neural cell fate”. Please see the text at line 369-370.

line354:  “ As shown in Fig. 6a-g” the figures meant appear to be 6c and g…?

Corrected

Line 397:  “form neural progenitors and neuronal cells with gene expression profiles similar to that of wild type ESCs (Fig. 6h, 7g)”. The levels are similar but more precisely, “slightly lower”
Response: corrected (line 425-428)

line 516: what is the neuro-significance of this Tg elavl3?

Response: Tg-elavl3: gfp (formerly HuC: gfp) enable us to directly visualize the HuC+ neurons by loss of Adnp. To make it clear, a reference paper is cited in the revised manuscript (line 462-463; referenced literature No. 42).

line 541:  “De novo mutations in ADNP have recently“ should read have been recently
Response: corrected (line 553)

Lines 552 to 555 states: ”These observations suggested that the intra-cellular localization of ADNP is tightly regulated during neural induction, or that ADNP plays an important role in the cytoplasm for the induction, maintenance and mature of neural cell types”. But in reality, these options are not mutually exclusive.
Response: the sentence was revised (line 616-619).

It is also curious that it is thought as a transcription factor, and it is found in the nucleus, so in addition to the epigenetic links described, one might still consider that it could modulate directly the expression of some unidentified targets.

Response: For the transcription factor role of ADNP, many developmental genes including the key neural genes and Wnt genes are likely to be indirectly regulated by ADNP (ADNP barely binds to these genes in ESCs, as discussed in the above). But we can not exclude the possibility that during neural differentiation ADNP is recruited and bind to these genes as a classic transcription factor. Also, ADNP may control gene expression by modulating chromatin structure. So we have made our conclusion more cautiously as you suggested (line591-608 in the revised manuscript).

Line 571: “exposing to toxins”…exposed to toxins.
Corrected (line 652)

Line 578” “we showed that ADNP-NAP alone can interact with β-Catenin, suggesting that NAP can execute similar function as ADNP in the regulation of Wnt/β-Catenin signaling pathway.” Given the overexpression and in vitro nature of the experiments for this portion, the statement should be much more cautious.
Response: the sentence was revised (line 659-662).

Line 614: “synaptic function and the chromatin remodeling.”
Response: the sentence was revised (line 713).

Line 621: “This may imply why ADNP is…” This may explain why

Corrected (line 720)

**Reviewer comments, second version:**

Reviewers' comments:

Reviewer #2 (Remarks to the Author):

The revised manuscript has improved but quantitative analyses remain as a major concern.
They have added qPCR and also quantification of cell differentiation. I still have concerns with quantitative analysis of western blotting. The quantification was performed using Photoshop. I have never seen anyone done quantification using this method. In the past, scientists have used Image-J or other quantitative image analysis software to quantify Western blot. More recently, quantitative analysis are done using equipment and software made for western (e.g. Li-Cor). The method used in this manuscript does not meet the scientific standard.
The method used for cellular quantitative analysis. The description (line 916-925) is confusing. ImageJ software was used, but it is unclear whether they are counting cell fluorescence intensity or number of positive cells. It is unclear how many “selected” fields were used, how many cells were sampled, what proportion of brain regions was sampled. The methods described are imprecise reflecting sloppiness of the investigators.


Reviewer #3 (Remarks to the Author):

The authors have modified considerably the original submission with comments and suggestions made, and this reviewer is satisfied with the new version of the manuscript.

**Author rebuttal, second version:**

Reviewers' comments:

Reviewer #2 (Remarks to the Author):

The revised manuscript has improved but quantitative analyses remain as a major concern.

Response: We thank the reviewer for the comments. We apologize for not properly describing the quantitative analyses. Please see details at lines 152-158; 169-176; 277-279;434-435 in the revised manuscript. For the quantitative method, please see details at lines 907-926; 1189-1196;1200-1204; 1209-1217; 1363-1366.

They have added qPCR and also quantification of cell differentiation. I still have concerns with quantitative analysis of western blotting. The quantification was performed using Photoshop. I have never seen anyone done quantification using this method. In the past, scientists have used Image-J or other quantitative image analysis software to quantify Western blot. More recently, quantitative analysis are done using equipment and software made for western (e.g. Li-Cor). The method used in this manuscript does not meet the scientific standard.

Response: we appreciate the reviewer for the comments for the WB quantification. We have used ImageJ for the quantification in the revised manuscript. (please see details at lines 856-862 of the revised manuscript)

The method used for cellular quantitative analysis. The description (line 916-925) is confusing. ImageJ software was used, but it is unclear whether they are counting cell fluorescence intensity or number of positive cells. It is unclear how many “selected” fields were used, how many cells were sampled, what proportion of brain regions was sampled. The methods described are imprecise reflecting sloppiness of the investigators. Response: First,we apologize for not clearly describing how we did the quantification in the previous manuscript. In the new revised manuscript, a clear and detailed information were provided, including how many fields selected; how many cells sampled, et al. (please see details at lines 907-926; 1189-1196;1200-1204; 1209-1217; 1363-1366).

We have compared both the methods: counting fluorescence intensity and number of positive cells. Basically, similar rescue effects were observed (Figure 7h shown as an example below), although they did exhibit difference (relative mean fluorescence intensity appears more sensitive than percentage of cells in this case).

As most of our IF experiments were performed using ESC-derived 3D neurospheres or aggregates. When dealing with the images of the neurospheres or aggregates, we found that it was difficult to accurately count cell numbers of an entire image. Selected area (with distinct cells) have to be used, in contrast to an entire image used for quantifying fluorescence intensity. Therefore, the number of cells used for calculating the percentage of positive cells (total cells less than 500) is usually less than that of quantifying fluorescence intensity (total cells more than 1000).

Based on above, we think that measuring fluorescence intensity might be a better choice. So, the relative fluorescence intensity method is primarily used and the results are shown in the revised manuscript.

Reviewer #3 (Remarks to the Author):

The authors have modified considerably the original submission with comments and suggestions made, and this reviewer is satisfied with the new version of the manuscript.

We thank this reviewer for help us improving the manuscript.

**Reviewer comments, third version:**

**Author rebuttal, third version:**
